# SLC-25A46 regulates mitochondrial fusion through the mitofusin protein FZO-1 and is essential for maintaining neuronal morphology

**DOI:** 10.1242/jcs.263571

**Published:** 2025-06-23

**Authors:** Hiroyuki Obinata, Taisei Watanabe, Hironori Takahashi, Satoshi Shimo, Toshiyuki Oda, Asako Sugimoto, Shinsuke Niwa

**Affiliations:** ^1^ Graduate School of Life Sciences, Tohoku University, Miyagi 980-8577, Japan; ^2^ Graduate School of Medicine, University of Yamanashi, Yamanashi 409-3898, Japan; ^3^ Department of Occupational Therapy, Health Science University, Yamanashi 401-0380, Japan; ^4^ Frontier Research Institute for Interdisciplinary Sciences (FRIS), Tohoku University, Miyagi 980-0845, Japan

**Keywords:** SLC25A46, Mitochondria, Mitofusin, Charcot–Marie–Tooth disease

## Abstract

Mitochondria are dynamic organelles shaped by sequential fission and fusion events. The mitochondrial protein SLC25A46 has been identified as a causative gene for mitochondrial neuropathies. However, the function of SLC25A46 in mitochondrial morphogenesis remains controversial, with several reports suggesting it acts as a mitochondrial fission factor, whereas others propose it as a fusion factor. In this study, employing forward genetics, we identified *slc-25A46*, a *Caenorhabditis elegans* ortholog of human SLC25A46, as an essential factor for mitochondrial fusion. Suppressor mutagenesis screening revealed loss-of-function mutations in *drp-1*, a mitochondrial fission factor, as suppressors of *slc-25A46*. The phenotype of *slc-25A46* mutants is similar to that of mutants in the worm mitofusin ortholog *fzo-1*, wherein the mitochondrial fusion factor is disrupted. Overexpressing FZO-1 mitigated mitochondrial defects in *slc-25a46* mutants, indicating that SLC-25A46 promotes fusion through FZO-1. Disease model worms carrying mutations associated with SLC25A46 exhibited mitochondrial fragmentation and accelerated neurodegeneration, suggesting that *slc-25A46* maintains neuronal morphology through regulating mitochondrial fusion regulation.

## Teaser

**Summary:** A forward genetic screen in *C. elegans* identify the mitochondrial protein SLC-25A46 as a mitochondrial fusion factor that regulates a mitofusin ortholog.

## INTRODUCTION

Mitochondria are highly dynamic organelles that constantly change their shapes to adapt to the metabolic states and physiological environments of the cell (Giacomello et al., 2020). The morphological diversities of mitochondria arise from sequential fission and fusion activities regulated by a family of GTPase enzymes (Giacomello et al., 2020). In humans, mitochondrial fission is mediated by dynamin-related protein 1 (DRP1, also known as DNM1L), a GTPase that forms ring-like structures at division sites (Antonny et al., 2016; Chappie et al., 2009). Conversely, mitochondrial fusion requires other GTPases, such as mitofusin 1 and 2 (MFN1 and MFN2) for outer membrane (OM) fusion and optic atrophy 1 (OPA1) for inner membrane (IM) fusion (Alexander et al., 2000; Westermann, 2008; Zuchner et al., 2004). In yeast, the fusion process involves the participation of another protein called Ugo1, which localizes to the OM and coordinates fusion between the outer and inner membranes by interacting with mitofusin and OPA1 orthologs (Sesaki and Jensen, 2001, 2004).

A mitochondrial protein SLC25A46, originally identified as a cause of optic atrophy and spastic paraplegia, shares weak similarities with Ugo1 (Abrams et al., 2015). Like Ugo1, SLC25A46 is localized on the OM, and forms a complex with MFNs and OPA1 (Janer et al., 2016). Despite these similarities, the exact function of SLC25A46 in mitochondrial dynamics remains controversial. Several studies have reported that acute knockdown of SLC25A46 in cultured cell lines leads to the formation of large elongated mitochondria (Abrams et al., 2015; Janer et al., 2016; Steffen et al., 2017). Knockdown of SLC25A46 has been shown to cause elongated mitochondria in zebrafish and *Drosophila* (Ali et al., 2020; Suda et al., 2018; Wan et al., 2016). A similar elongated mitochondrial phenotype has been observed in SLC25A46 mutant mice that have a 46-bp deletion in the exon 8 (Li et al., 2017). Conversely, overexpression of SLC25A46 induces mitochondrial fragmentation (Abrams et al., 2015). All of these studies support the function of SLC25A46 as a fission factor. In contrast, the complete loss of SLC25A46 in the knockout HeLa cell results in the fragmentation of the mitochondrial network (Schuettpelz et al., 2023). In a separate study using a knockout mouse model, loss of SLC25A46 function led to the formation of small mitochondrial fragments in the nervous system (Duchesne et al., 2017). A mutation in the bovine *SLC25A46* gene is associated with sensorimotor neuropathy, known as Turning calves syndrome, wherein fragmented and aggregated mitochondria were observed (Duchesne et al., 2017). Moreover, a null allele of the human *SLC25A46* gene has been identified as a cause of optic atrophy spectrum disorder (Nguyen et al., 2017). In cells from individuals with this variants, mitochondrial fragmentation was observed (Nguyen et al., 2017). These phenotypes are similar to those observed in yeast Ugo1 mutants, suggesting that primary role of SLC25A46 is as a mitochondrial fusion factor.

*Caenorhabditis elegans* is a widely-used model organism for studying organelle dynamics and morphogenesis, including the nucleus, synaptic vesicles and mitochondria (Campbell and Zuryn, 2024; Ichishita et al., 2008; Meyerzon et al., 2009; Niwa et al., 2016; Ren et al., 2023; Zhao et al., 2021). Disruption of intracellular transport of mitochondria is observed in *unc-116*, *miro-1* and *trak-1* mutant worms, where the molecular motor kinesin-1 and its adaptor proteins are mutated (Ren et al., 2023; Zhao et al., 2021). Orthologs of these proteins have similar functions in mammals and flies (Campbell and Zuryn, 2024). Mitochondrial fragmentation has been reported in *fzo-1* and *eat-3* mutant worms (Rolland et al., 2009). *fzo-1* and *eat-3* respectively encode mitofusin (MFN1 and MFN2), and OPA1 orthologs. By contrast, mitochondrial hyperfusion has been observed in *drp-1* mutants, in which a DRP1 ortholog is disrupted (Labrousse et al., 1999; Scholtes et al., 2018).

In this study, through forward genetic screening in *C. elegans*, we identified a loss-of-function mutation in *slc-25A46* gene, a *C. elegans* ortholog of human *SLC25A46*. The *slc-25A46* mutant worms exhibited fragmented and small mitochondria, similar to the phenotype observed in worms lacking the worm mitofusin *fzo-1*. Notably, this defect was partially rescued by a loss-of-function mutation in *drp-1* or by overexpressing *fzo-1*. These findings suggest that *slc-25A46* is upstream of *fzo-1* and is essential for mitochondrial fusion, rather than fission. To further investigate the function of SLC25A46, we introduced pathogenic variants reported in the human *SLC25A46* gene into *C. elegans slc-25A46* gene and examined their effects on mitochondrial morphology and distribution. Our results indicate that *slc-25A46* mutations accelerated the morphological degeneration in neurons. Collectively, these findings suggest that *slc-25A46* plays a crucial role in mitochondrial morphology and neuronal maintenance.

## RESULTS

### Isolation of *slc-25A46* mutants

**Fig. 1.**
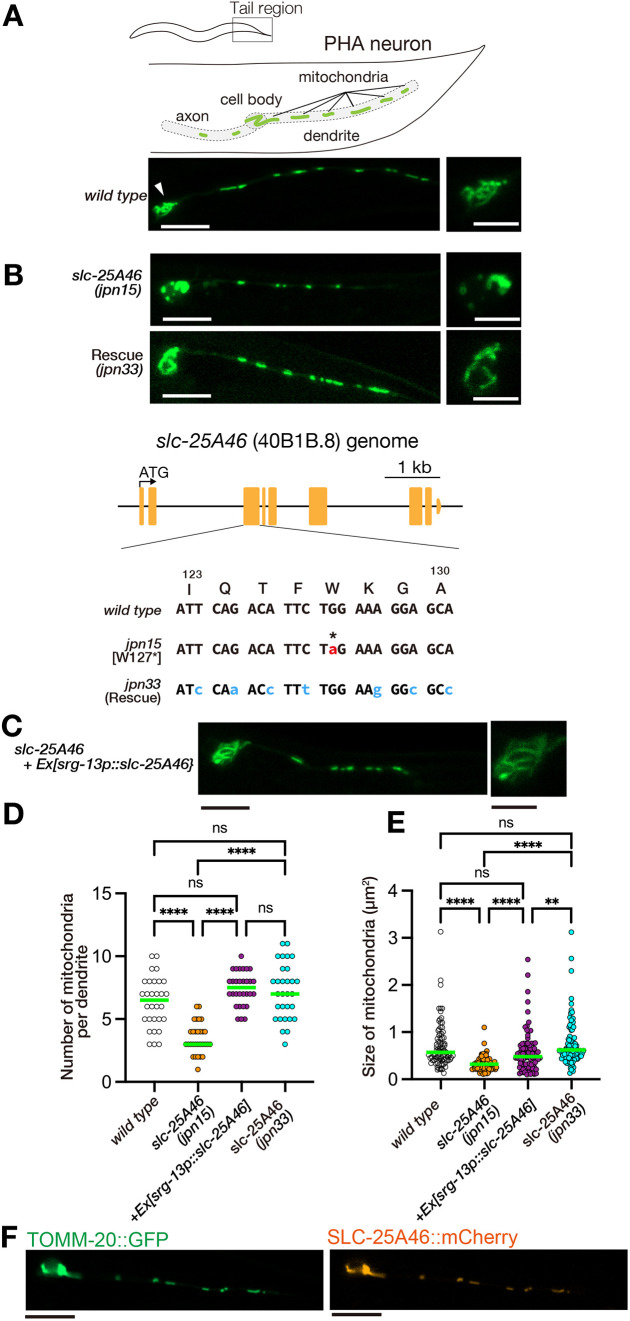
**Identification of *slc-25A46* mutant.** (A) Schematic representation of PHA neuron morphology (top) and corresponding fluorescence image showing the morphology and distribution of mitochondria in PHA neuron (bottom). The arrowhead marks the cell body, which is also shown separately in the left panel. A mitochondrial targeting sequence from TOMM-20 (residue 1–54) was fused to GFP and expressed under the *flp-15* promoter. Scale bars: 10 µm (left); 5 µm (right). (B) Alleles of *slc-25A46* and their impact on mitochondrial morphology. The *jpn15* allele, isolated through EMS mutagenesis, has a nonsense mutation (W127Ter) in the *slc-25A46* gene and exhibits fragmented mitochondria phenotype in the PHA neuron. Using CRISPR/Cas9 technology, the nonsense mutation in the *slc-25A46(jpn15)* allele was corrected. The resultant allele *slc-25A46(jpn33)* has normal mitochondria. Scale bars: 10 µm (left); 5 µm (right). (C) Representative image showing the cell-autonomous rescue in *slc-25A46* mutants. *slc-25A46* cDNA was expressed under the control of the PHA neuron-specific *srg-13* promoter. Scale bars: 10 µm (left); 5 µm (right). Tubular mitochondria are evident in both the dendrite and the cell body. Images in A–C are representative of at least 50 independent worms per genotype. (D) Dot plots showing the number of mitochondria in the PHA dendrite. Each dot shows the number of mitochondria in a single PHA dendrite. Green bars represent median values. *n*=30 dendrites from 30 worms. ****P<0.0001; ns, not significant (*P*>0.05) (Kruskal–Wallis test followed by Dunn's multiple comparison test). (E) Dot plots showing the size distribution of mitochondria in the PHA dendrite. Each dot represents the size of an individual mitochondrion in the PHA dendrite. Green bars represent median values. *n*=85 mitochondria.. ****P<0.0001; **P<0.01; ns, not significant (*P*>0.05) (Kruskal–Wallis test followed by Dunn's multiple comparison test). (F) Representative images showing the localization of SLC-25A46::mCherry in PHA neurons. SLC-25A46::mCherry (orange) was expressed under the control of the *srg-13* promoter. TOMM-20::GFP (green) was used as a mitochondrial marker. Scale bars: 10 µm. Images representative of at least 20 independent observations from two lines.

It is established that mitochondria accumulate in dendrites of ciliated sensory neurons, such as olfactory neurons (Reese, 1965). To elucidate the molecular mechanisms of mitochondrial morphogenesis in sensory dendrites, we labeled mitochondria in *C. elegans* PHA neurons (Fig. 1A). GFP was fused to the N-terminal 54 amino acids of the TOMM-20 protein and used as a marker to visualize mitochondria (Ichishita et al., 2008; Kanaji et al., 2000). The PHA neuron is a highly polarized neuron characterized by a dendrite with sensory cilia, a cell body and an axon (Inglis et al., 2006). We utilized the *flp-15* promoter, which exclusively expresses in PHA neurons (Niwa, 2016). In the resulting strain, intense GFP fluorescence of mitochondria was observed in both the soma and dendrites (Fig. 1A). However, analyzing signals in the axon was challenging due to the sparse distribution of mitochondria and the autofluorescence of gut cells.

To identify molecules essential for mitochondrial morphogenesis, we conducted forward genetic screens through genome-wide ethyl methanesulfonate (EMS) mutagenesis (Brenner, 1974). We isolated a mutant allele named *jpn15*. In *jpn15* worms, the number of mitochondria was reduced, and their size was smaller compared to that seen in wild-type worms (Fig. 1B). Through genetic mapping and genome sequencing, we identified a nonsense mutation at W127 of the *slc-25A46* gene, which is the *C. elegans* ortholog of the mammalian SLC25A46 (Fig. 1B). A previous RNAi screen of mitochondrial morphology did not link the function of s*lc-25A46* with mitochondrial morphogenesis (Ichishita et al., 2008). To confirm that the W127Stop mutation in the *slc-25A46* gene is the causative mutation for the *jpn15* phenotype, we used CRISPR/Cas9-mediated genome editing to replace the nonsense mutation in *jpn15* with the wild-type amino acid. We introduced silent mutations and created a restriction enzyme site in the repair template to prevent subsequent Cas9 cleavage and verify successful genome editing (Fig. 1B). The resultant allele, *jpn33*, carrying the repaired *slc-25A46*, showed restored mitochondrial number and size, indicating that the W127Stop mutation in *slc-25A46* locus is responsible for the abnormal mitochondrial morphology observed in *jpn15* allele (Fig. 1B). To test whether SLC-25A46 functions in a cell-autonomous manner, *slc-25A46(cDNA)* was expressed under the control of the PHA neuron-specific promoter, *srg-13* promoter (*srg-13p*) (Fig. 1C) (Niwa, 2016). In transgenic animals carrying extrachromosomal arrays that expressed *slc-25A46(cDNA)*, both mitochondrial number and morphology were restored to wild-type levels (Fig. 1C). To quantify the mitochondrial morphology, we measured both the number and size of mitochondria in PHA dendrites (Fig. 1D,E). Statistical analysis confirmed that loss of *slc-25A46* significantly reduced mitochondrial number and size in PHA dendrites. Both correction of the mutation by CRISPR/Cas9 and expression of *slc-25A46* cDNA restored these parameters to wild-type levels. SLC-25A46::mCherry localized to mitochondria (Fig. 1F), showing a uniform distribution without regional enrichment. Additionally, we found that *slc-25A46(gk570223)*, obtained from the ‘million mutation’ project (Thompson et al., 2013), had a Q310Stop mutation and showed the mitochondrial fragmentation phenotype (Fig. S1A).

Next, to determine whether mitochondrial fragmentation occurs in other tissues of *slc-25A46(jpn15)* mutant worms, we observed mitochondria within the body wall muscles. Similar to the observations in the PHA neuron cell bodies, mitochondria in *slc-25A46(jpn15)* mutants exhibited rounded and fragmented structures compared to wild type (Fig. S1B). Prior studies have shown that cristae structures are degenerated by SLC25A46 mutations in human and mice (Janer et al., 2016; Li et al., 2017; Schuettpelz et al., 2023), whereas another study has shown that cristae structures are not strongly affected in SLC25A46-knockout mice (Duchesne et al., 2017). We observed the fine structure of mitochondria in the muscular cells by transmission electron microscopy. Our observation suggested that cristae structures in mitochondria was not strongly affected in *slc-25A46* mutant worms (Fig. S1C,D), resembling the findings described in Duchesne et al. (2017).

Overall, our findings suggest that *slc-25A46* plays a crucial role in maintaining the size and distribution of mitochondria.

### *drp-1* mutation partially suppresses mitochondrial fragmentation in *slc-25A46* mutants

**Fig. 2.**
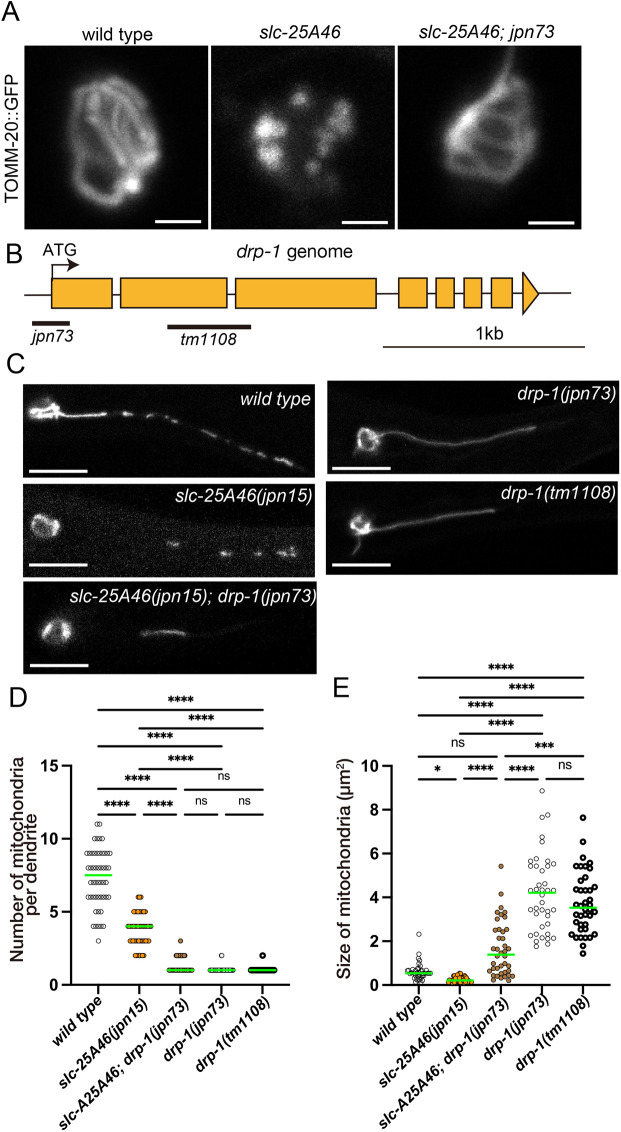
**SLC-25A46 and DRP1 have opposite functions in mitochondrial morphogenesis.** (A) Identification of *jpn73*, a suppressor mutant of the *slc-25A46* mutant. Representative images showing mitochondria in the cell body of *wild type*, *slc-25A46*, *slc-25A46*; *jpn73* mutants. Note that fragmented mitochondria phenotype in *slc-25A46* is suppressed in *slc-25A46; jpn73* double mutant. Scale bars: 2 µm. (B) Schematic drawing showing the genome structure of *drp-1* gene and the *jpn73* mutation. Deleted regions in *jpn73* and *tm1108* alleles are shown by bars. (C) Representative images showing the mitochondrial morphology in the dendrite of wild type, *slc-25A46(jpn15)*, *slc-25A46(jpn15); drp-1(jpn73)*, *drp-1(jpn73)* and *drp-1(tm1108)*. Scale bars: 10 µm. Images in A and C are representative of at least 50 independent worms per genotype. (D) Dot plots showing the number of mitochondria in the PHA dendrite. Each dot shows the number of mitochondria in a single PHA dendrite. Green bars represent median values. *n*=50 dendrites from 50 worms. ****P<0.0001; ns, not significant (*P*>0.05) (Kruskal–Wallis test followed by Dunn's multiple comparison test). (E) Dot plots showing the size distribution of mitochondria in the PHA dendrite. Each dot represents the size of an individual mitochondrion in the PHA dendrite. Green bars represent median values. *n*=40 mitochondria. *P<0.05; ***P<0.001; ****P<0.0001; ns, not significant (*P*>0.05) (Kruskal–Wallis test followed by Dunn's multiple comparison test).

To understand the function of SLC25A46 in mitochondrial morphogenesis, we performed a second EMS mutagenesis screens and searched for mutations that could suppress the mitochondrial fragmentation phenotype in the *slc-25A46(jpn15)* mutant. A mutant allele, named *jpn73*, could recover the mitochondrial network in the cell body and dendrite of *slc-25A46(jpn15)* mutant (Fig. 2A–E). Mapping and sequencing revealed that *jpn73* is a mutant allele of *drp-1* (Fig. 2B). Genome sequencing revealed that exon 1 of the *drp-1* gene, including the start codon, was deleted in the *drp-1(jpn73)* allele (Fig. 2B). DRP-1 is a *C. elegans* ortholog of DRP1 that is essential for the mitochondrial fission (Labrousse et al., 1999; Scholtes et al., 2018). We next compared the dendritic phenotype of *drp-1(jpn73)*, *slc-25A46(jpn15)* and *slc-25A46(jpn15)*; *drp-1(jpn73)* mutants (Fig. 2C–E). Moreover, we observed the phenotype of *drp-1(tm1108)* allele, which is a null allele of *drp-1*, for comparison (Fig. 2B). In the dendrite of PHA neuron, both *drp-1(jpn73)* and *drp-1(tm1108)* mutants had an elongated mitochondrion, indicating mitochondrial hyperfusion (Fig. 2C). Compared to *slc-25A46* mutants, *slc-25A46; drp-1(jpn73)* double mutants exhibited larger mitochondria, and showed a reduced number of mitochondria (Fig. 2C–E), indicating that *drp-1(jpn73)* mutation either induces mitochondrial fusion or reduces mitochondrial fission. The phenotype of *drp-1* mutant worms was clearly different from mitochondrial fragmentation phenotype observed in *slc-25A46(jpn15)* mutant worms (Figs 1 and 2C). These results shows that SLC-25A46 and DRP-1 have opposite functions in the mitochondrial morphogenesis. As DRP-1 is a mitochondrial fission factor, these data suggested that SLC-25A46 works as a mitochondrial fusion factor.

### *slc-25A46* works as a mitochondrial fusion factor in *C. elegans*

**Fig. 3.**
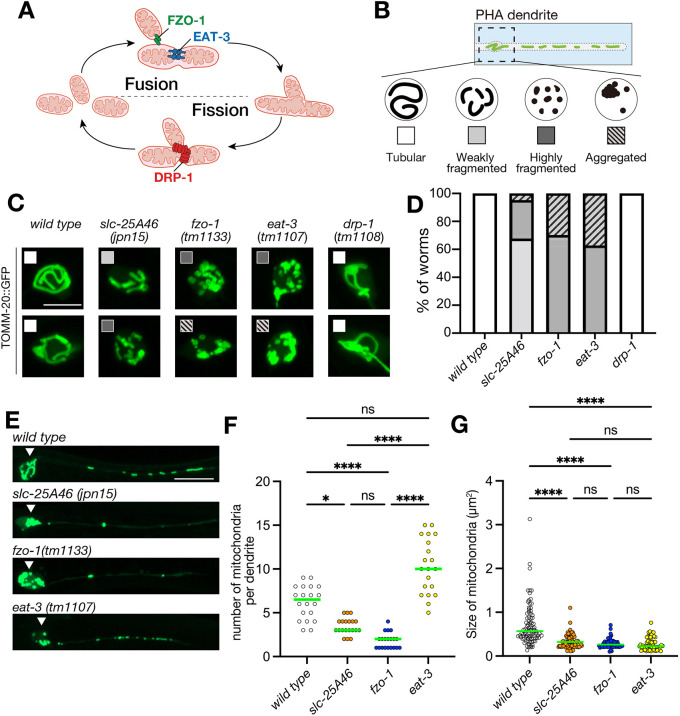
**Comparison of mitochondrial phenotypes.** (A) Schematic drawing showing the mitochondrial dynamics and functions of FZO-1 (a mitofusin ortholog), EAT-3 (a OPA1 ortholog) and DRP-1 (a DRP1 ortholog). (B) Schematic drawing showing the category of mitochondrial morphology used in this study. (C,D) Mitochondrial morphology in the cell body of PHA neuron. Representative images showing the mitochondrial morphology in PHA cell body and their categories (C). In D, a stacked bar graph illustrates the ratio of categories in each mutant. *N*=40 worms. Scale bars: 2 µm. (E) Representative images showing mitochondrial morphology in the PHA neuron. Arrowhead highlights the cell body. Images representative of at least 50 independent worms per genotype. Scale bars: 10 µm. (F) Dot plots showing the number of mitochondria in the PHA dendrite. Each dot shows the number of mitochondria in a single PHA dendrite. Green bars represent median values. *n*=20 dendrites from 20 worms. *P<0.05. ****P<0.0001; ns, not significant (*P*>0.05) (Kruskal–Wallis test followed by Dunn's multiple comparison test). (G) Dot plots showing the size distribution of mitochondria in the PHA dendrite. Each dot represents the size of an individual mitochondrion in the PHA dendrite. Green bars represent median values. *n*=85, 83, 53 and 60 mitochondria for each genotype. ****P<0.0001; ns, not significant (*P*>0.05) (Kruskal–Wallis test followed by Dunn's multiple comparison test).

To study the phenotype of *slc-25A46*, we performed a comparative analysis of mitochondrial morphology using *C. elegans* mutants with loss-of-function mutations in key genes associated with mitochondrial dynamics, including *drp-1* (Fig. 2), *fzo-1* and *eat-3* (Fig. 3A). FZO-1 and EAT-3, orthologs of mitofusin (MFN1 and MFN2) and OPA1 respectively, play important roles in mitochondrial fusion (Fig. 3A). We first focused on the morphology of mitochondria within the cell bodies of PHA neurons. Mitochondria were categorized into tubular, fragmented, and aggregated forms (Fig. 3B and see Materials and Methods). Within the fragmented category, a further distinction was made between weak and strong fragmentation based on the size of the fragmented mitochondria (Fig. 3B and Materials and Methods). In the cell body of wild type and *drp-1(tm1108)* mutants, we did find a tubular mitochondrial network (Fig. 3C,D). No fragmented mitochondria were observed in the wild type, nor in *drp-1(tm1108)* mutants. In contrast, the cell body of the *slc-25A46(jpn15)* mutant contained fragmented and small mitochondria, similar to the cell bodies of *fzo-1(tm1133)* and *eat-3(tm1107)* mutants (Fig. 3C,D). However, the degree of fragmentation in *slc-25A46(jpn15)* was milder than that in *fzo-1(tm1133)* and *eat-3(tm1107)* mutants (Fig. 3D). Next, we compared the number and morphology of mitochondria in the PHA dendrite. In *slc-25A46(jpn15)* mutants*,* there was a significant decrease in both the number and size of mitochondria compared to that in the wild type (Fig. 3E–G). Similarly, *fzo-1(tm1133)* mutants displayed reductions in both the number and size of mitochondria. Interestingly, *eat-3(tm1107)* mutants only exhibited a decrease in mitochondrial size, without abnormal localization*.* Based on these phenotypes, we suggest that SLC-25A46 acts as a fusion factor for mitochondria, similar to FZO-1 and EAT-3. Notably, the phenotype of *slc-25A46* is more similar to that of *fzo-1* than to that of *eat-3*.

### Ectopic expression of FZO-1 partially suppresses defects in the *slc-25a46* mutant

**Fig. 4.**
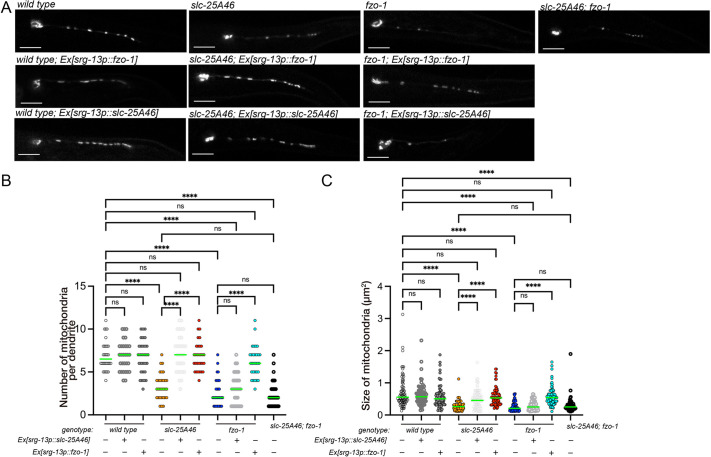
**FZO-1 is downstream of SLC-25A46.** (A) Representative images showing the mitochondrial morphology in PHA neurons of wild type, *slc-25A46(jpn15)* and*fzo-1* single mutants and *slc-25A46; fzo-1* double mutants, as well as strains overexpressing FZO-1 or SLC-25A46 in each mutant background. Note that expression of SLC-25A46 rescued mitochondrial defects in *slc-25A46* but not in *fzo-1* mutants, whereas overexpression of FZO-1 restored mitochondrial morphology in both *slc-25A46(jpn15)* and *fzo-1* mutants. Images representative of at least 20 independent worms per genotype. Scale bars: 10 µm. (B) Dot plots showing the number of mitochondria in the PHA dendrite. Each dot shows the number of mitochondria in a single PHA dendrite. Green bars represent median values. *n*=30 dendrites from 30 worms. ****P<0.0001; ns, not significant (*P*>0.05) (Kruskal–Wallis test followed by Dunn's multiple comparison test). (C) Dot plots showing the size distribution of mitochondria in the PHA dendrite. Each dot represents the size of an individual mitochondrion in the PHA dendrite. Green bars represent median values. *n*=80 mitochondria, respectively. ****P<0.0001. ns, not significant (*P*>0.05) (Kruskal–Wallis test followed by Dunn's multiple comparison test).

The mammalian SLC25A46 has been reported to physically interact with the outer membrane fusion factors MFN1 and MFN2 (FZO-1 orthologs) (Steffen et al., 2017). The above genetic experiments suggest that *slc-25A46* and *fzo-1* work together in mitochondrial morphogenesis. However, the functional interaction between SLC25A46 and MFNs was still elusive. To investigate the relationship further, we conducted additional genetic experiments. First, we generated *slc-25A46*; *fzo-1* double mutants and found that the phenotype was not enhanced compared with that in either the *slc-25A46* or *fzo-1* single mutants (Fig. 4A–C), suggesting that *slc-25A46* and *fzo-1* work in the same genetic pathway We next expressed FZO-1 in the *slc-25A46* mutant using the PHA-specific *srg-13* promoter (Fig. 4A–C). Such ectopic expression of FZO-1 in PHA neurons was able to restore both the number and size of mitochondria in *slc-25A46(jpn15)* mutant (Fig. 4A–C)*.* In a wild-type background, the expression of FZO-1 did not enhance mitochondrial fusion. In addition, we expressed SLC-25A46 in mutant backgrounds. Whereas *srg-13p::slc-25A46* could rescue the phenotype of *slc-25A46* mutants, the same extrachromosomal array could not rescue the phenotype of *fzo-1* mutants (Fig. 4A–C). These results suggest that *slc-25A46* and *fzo-1* work in the same pathway to determine mitochondrial morphology and that *slc-25A46* is genetically upstream of *fzo-1*.

### Overexpression of SLC-25A46 induces mitochondrial fragmentation

**Fig. 5.**
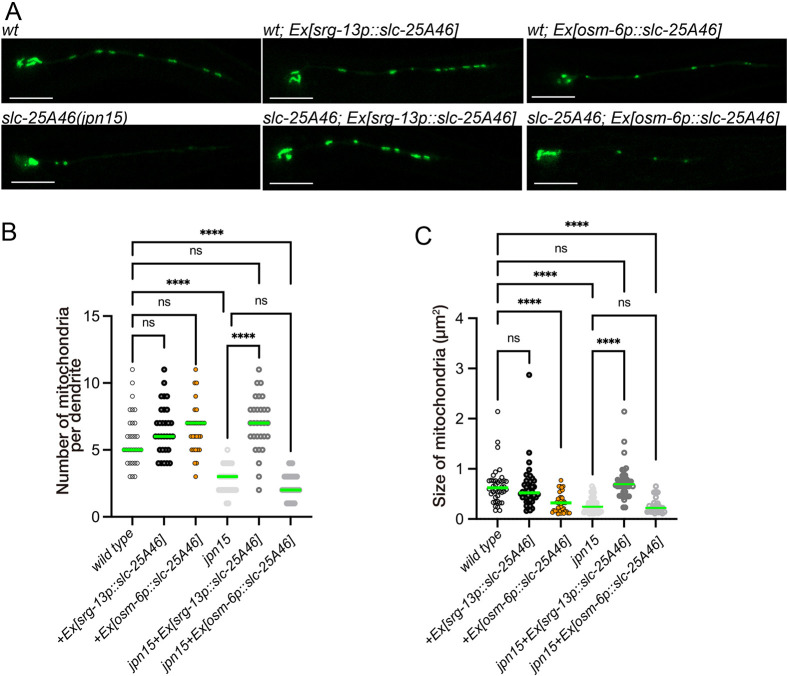
**Overexpression of *slc-25A46* induces mitochondrial fragmentation.** (A) Representative images of mitochondrial morphology in PHA neurons of wild-type animals, wild-type animals expressing *slc-25A46* under the control of the *srg-13* or *osm-6* promoter, *slc-25A46* mutant animals, and *slc-25A46* mutants expressing *slc-25A46* driven by the *srg-13* or *osm-6* promoter. Mitochondria were visualized using TOMM-20(1-54 a.a)::GFP. Expression of *slc-25A46* under the *srg-13* promoter restored normal mitochondrial morphology in mutants, whereas expression under the *osm-6* promoter failed to rescue. Notably, *osm-6*-driven expression of *slc-25A46* in *wild-type* background induced mitochondrial fragmentation. Images representative of at least 20 independent worms per genotype. Scale bars: 10 µm. (B) Dot plots showing the number of mitochondria in the PHA dendrite. Each dot represents the number of mitochondria in a single PHA dendrite. Green bars indicate median values. *n*=30 dendrites from 30 animals. ****P<0.0001; ns, not significant (*P*>0.05) (Kruskal–Wallis test followed by Dunn's multiple comparison test). (C) Dot plots showing the size distribution of mitochondria in the PHA dendrite. Each dot represents the size of an individual mitochondrion in the PHA dendrite. Green bars represent median values. *n*=40 mitochondria, respectively. ****P<0.0001; ns, not significant (*P*>0.05) (Kruskal–Wallis test followed by Dunn's multiple comparison test).

During the rescue experiment, we noticed that expression of *slc-25A46* under the control of the *osm-6* promoter, rather than the *srg-13* promoter, induced mitochondrial fragmentation in a wild-type background (Fig. 5A–C). This result is consistent with previous studies demonstrating that overexpression of SLC25A46 leads to mitochondrial fragmentation in zebrafish and mammalian cells (Abrams et al., 2015). However, this observation appears to contradict the loss-of-function phenotypes, which are also mitochondrial fragmentation (Fig. 1) (Nguyen et al., 2017; Schuettpelz et al., 2023). To address this contradiction, we expressed *slc-25A46* cDNA under the *srg-13* or *osm-6* promoter in the *slc-25A46(jpn15)* mutant background. Whereas expression under the *srg-13* promoter could rescue the mitochondrial morphology, expression from the *osm-6* promoter failed to rescue the mutant (Fig. 5A–C). These observations support the interpretation that SLC-25A46 is a mitochondrial fusion factor, but overexpression, particularly at high levels, can itself induce mitochondrial fragmentation. To test whether the phenotype is associated with differences in promoter strength, we compared expression levels of *osm-6* and *srg-13* promoter by quantifying mean GFP fluorescent intensity in PHA neurons of transgenic animals carrying *osm-6p::gfp* or *srg-13p::gfp* reporter extrachromosomal arrays (Table S3, OTL336-339). When the fluorescent intensity of GFP were measured in PHA neurons, the expression level of the *osm-6* promoter was ∼8.5-fold higher than that of the *srg-13* promoter (mean±s.d.: 35,027±5289 for *osm-6p::gfp* and 4159±961 for *srg-13p::gfp. P*<0.001, unpaired two-tailed *t*-test. *n*=20 neurons from two independent transgenic lines per genotype). These results are consistent with our hypothesis that overexpression of *slc-25A46* at high levels induces mitochondrial fragmentation.

### SLC25A46-associated disease model worms

**Fig. 6.**
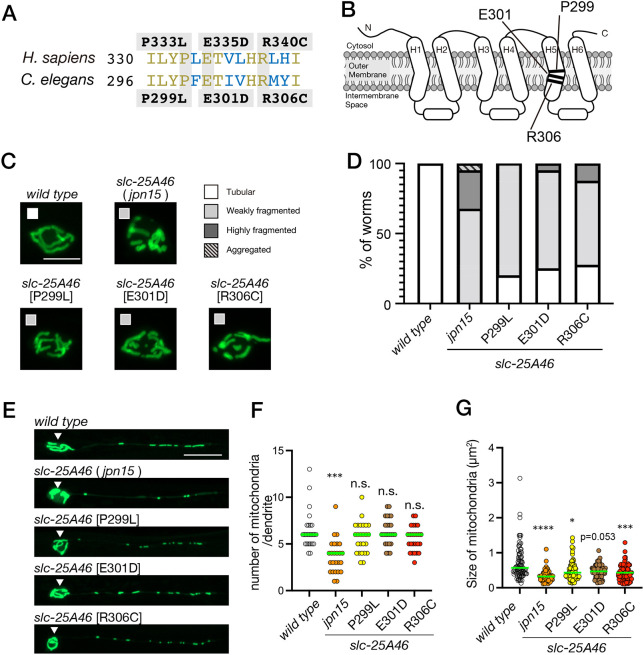
**Establishment of SLC25A46-associated disease models.** (A) A sequence comparison between human SLC25A46 and *C. elegans* SLC-25A46. Three amino acid changes associated with SLC25A46-related diseases (P333L, E335D and R340C) and the corresponding amino acid changes in *C. elegans*, analyzed in this study, are illustrated. (B) Schematic drawing showing the predicted structure of *C. elegans* SLC-25A46 and mutations analyzed in this study. (C,D) Mitochondrial morphology in the cell body of PHA neuron. Representative images showing the mitochondrial morphology in PHA cell body in *slc-25A46* alleles and their categories (C). In D, a stacked bar graph illustrates the ratio of categories in each mutant. Mitochondrial morphology was categorized using the criteria shown in Fig. 3B. *N*=40 worms. Scale bar: 2 µm. (E) Representative images showing the mitochondrial morphology in the PHA neuron of wild-type and *slc-25A46* mutant alleles. Arrowheads indicate the cell body. Images representative of at least 50 independent worms per genotype. Scale bar: 5 µm. (F) Dot plots showing the number of mitochondria in the PHA dendrite. Each dot shows the number of mitochondria in a single PHA dendrite. Green bars represent median values. *n*=20 dendrites from 20 worms for each genotype. ***P<0.001; ns, not significant (*P*>0.05) (Kruskal–Wallis test followed by Dunn's multiple comparison test). (G) Dot plots showing the size distribution of mitochondria in the PHA dendrite. Each dot represents the size of an individual mitochondrion in the PHA dendrite. Green bars represent median values. *n*=85, 83, 83, 80, 80 and 80 mitochondria for each genotype, respectively. * *P*<0.05; ***P<0.001; ****P<0.0001; actual *P*-value is shown for *slc-25A46(E301D)* mutant (Kruskal–Wallis test followed by Dunn's multiple comparisons test).

Human *SLC25A46* has been identified as a causative gene for neurodegenerative disorders, such as Charcot–Marie–Tooth disease and optic atrophy (Abrams et al., 2015). Most of these diseases are caused by autosomal recessive mutations, and numerous amino acid changes have been reported as disease-causing in humans. However, the mechanisms underlying disease onset remain unclear. Therefore, we attempted to validate the effects of disease-causing mutations on mitochondrial morphology and distribution by introducing these mutations into the *C. elegans slc-25A46* gene. Among the human disease-associated variants reported to date, we introduced three mutations into *C. elegans slc-25A46* gene by CRISPR/Cas9 (Fig. 6A). These residues are embedded in the OM (Fig. 6B). First, we observed the mitochondrial morphology in the cell body of mutant alleles. We found mitochondria are fragmented in disease-associated *slc-25A46* mutant worms (Fig. 6C,D). However, compared with *slc-25A46(jpn15)*, which is considered to be a null allele, these three mutants showed more subtle mitochondrial fragmentation in the cell body (Fig. 6C,D). Next, we observed the distribution and mitochondrial morphology in the PHA dendrite (Fig. 6E–G). The size of mitochondria was statistically smaller in two out of three disease mutant model worms (Fig. 6G). Although we could not detect statistical significance, *slc-25A46(E301D)* also showed a tendency towards having smaller mitochondria. In contrast, the number of mitochondria was not significantly affected by these disease-associated mutations (Fig. 6F). These data suggest that disease-associated SLC25A46 mutations result in partial loss of function in the regulation of mitochondrial fusion event.

### The neuronal morphology is disrupted by *slc25-a46* mutations

**Fig. 7.**
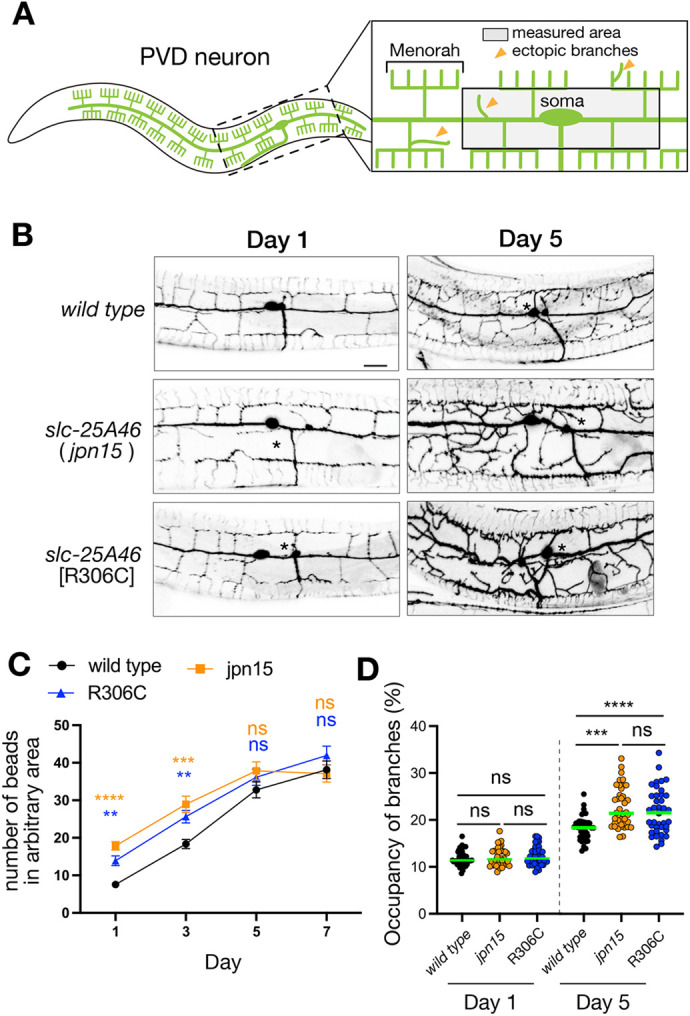
**Neuronal morphology in *slc-25A46* mutant worms.** (A) Schematic drawing of the morphology of the PVD neuron. The drawing also illustrates examples of ectopic branches. Measured area in C and D is shown by a gray box. (B) Representative images showing the morphology of PVD neurons in wild-type and *slc-25A46* mutant alleles at deay 1 and 5. Asterisks indicate ectopic branches. Images representative of at least 20 independent worms per genotype. Scale bar: 10 µm. (C) A graph showing the number of bead-like structures along the dendrite of PVD neuron at days 1, 3, 5 and 7. Measured area is as indicated in A. Mean±s.e.m. is indicated. *n*=20 worms for each genotype and each day. **P<0.01; ***P<0.001; ****P<0.0001; ns, not significant (*P*>0.05) (one-way ANOVA followed by Dunnett's multiple comparisons test was performed using wild type as a control). (D) Dot plots showing the percentage of area occupied by PVD neurons within the region illustrated in panel A. An elevated value is considered to represent an increase in ectopic branches. Mean±s.e.m. is indicated. *n*=40 PVD neurons from 40 worms for each genotype and each day. ***P<0.001; ****P<0.0001; ****P<0.0001; ns, not significant (*P*>0.05) (one-way ANOVA followed by Dunnett's multiple comparisons test).

Mutations in the human *SLC25A46* gene have been linked to neuronal degeneration (Abrams et al., 2015; Janer et al., 2016). In light of this, we investigated the neuronal morphology in our *slc-25A46* mutant worms. Specifically, we observed the morphology of PVD neurons, a model neuronal cell often used to study neurodegeneration and regeneration in *C. elegans* (Brar et al., 2022; Oren-Suissa et al., 2017; Zhao et al., 2021) (Fig. 7A). First, we analyzed mitochondrial morphology in PVD neurons (Fig. S2). Similar to PHA neurons, PVD neurons in *slc-25A46(jpn15)* and *slc-25A46(R306C)* mutant backgrounds exhibited abnormal mitochondrial morphology (Fig. S2A–C). The number and size of mitochondria in *slc-25A46(jpn15)* and *slc-25A46(R306C)* PVD neurons showed similar alterations to those observed in PHA neurons (Fig. S2B,C). Mitochondrial defects in *slc-25A46(jpn15)* mutants could be rescued by the expression of *slc-25A46* cDNA under the control of *per-2* promoter, a PVD-specific promoter (Dong et al., 2013) (Fig. S2B,C), suggesting that *slc-25A46* works in PVD neurons to regulate mitochondrial morphology. Next, we observed adult-stage worms from day 1 to day 7 after the final molting. In 1-day-old adult worms, the overall morphology of the PVD neuron appeared to be unaffected by *slc-25A46* mutations, preserving the characteristic menorah structure (Dong et al., 2013) (Fig. 7A,B). However, we noticed an increased number of bead-like structures along the dendrite in *slc-25A46* mutants at day 1 and day 3 (Fig. 7B). Bead-like structures, a reported sign of neuronal degeneration (Nakano et al., 2022; Oren-Suissa et al., 2017; Zhao et al., 2021), are defined in this study as regions where the dendrite diameter is more than three times that of the adjacent shaft. The number of such structures was manually counted along the dendrite in each neuron, revealing a significant increase in *slc-25A46* mutants at day 1 and day 3 (Fig. 7C). In 5- and 7-day-old adult worms, because the number of bead-like structures is increased even in wild-type worms, no significant difference was observed between wild-type and *slc-25A46* mutants (Fig. 7C). However, the morphological defects were more evident in *slc-25A46* mutant worms at day 5, showing increased numbers of ectopic branches (Fig. 7B). To quantify the increase in ectopic dendritic branches, we measured the relative area occupied by neuronal processes within a defined region surrounding the PVD cell body (Fig. 7D). Specifically, we defined a 100 µm×Y µm rectangular area centered on the soma, where the Y-dimension was bounded by the adjacent tertiary branches (Fig. 7A, gray area). This parameter was used instead of direct branch counting, as distinguishing individual ectopic branches from normal higher-order dendrites was ambiguous owing to their irregular orientation and morphology. We found that the area occupied by neurites was significantly increased in 5-day-old *slc-25A46* mutants compared to age-matched wild-type animals (Fig. 7D). Previous studies have shown that ectopic branches of PVD neurons increase when neurons are injured and fail to be repaired (Brar et al., 2022; Oren-Suissa et al., 2017). These data indicate that *slc-25A46* mutations accelerate morphological degeneration in neurons.

## DISCUSSION

### SLC-25A46 is essential for mitochondrial fusion

The functional role of SLC25A46 remains controversial, as previous studies have reported inconsistent mitochondrial phenotypes across different models. Mitochondrial hyperfusion has been observed in knockdown models of zebrafish and *Drosophila* (Suda et al., 2018; Wan et al., 2016), whereas both hyperfusion and fragmentation have been reported in mammalian systems depending on the mode of genetic manipulation (Abrams et al., 2015; Janer et al., 2016; Li et al., 2017; Steffen et al., 2017). These findings suggest that mitochondrial morphology in response to SLC25A46 loss might depend not only on the degree of gene disruption, but also on context-dependent factors. In our *C. elegans* model, the *slc-25A46(jpn15)* allele obtained through unbiased genetic screen, which has an early stop codon and is likely a null mutation, led to mitochondrial fragmentation. We found that the mitochondrial phenotype of *slc-25A46* mutants was similar to those of *fzo-1* and *eat-3* mutants. FZO-1 is an ortholog of MFN1 and MFN2, whereas EAT-3 is an ortholog of OPA1; both are essential factors for mitochondrial fusion. Consistent with the role of SLC-25A46 in mitochondrial fusion, suppressor mutagenesis screening on *slc-25A46* mutant background shows that a *drp-1* mutation, which disrupts the function of mitochondrial fission factor, can antagonize the phenotype of *slc-25A46*. These genetic data suggest that *slc-25A46* is required for the mitochondrial fusion, rather than fission. Furthermore, mitochondrial number is more severely reduced in *slc-25A46* and *fzo-1* mutants than in *eat-3* mutants. The phenotypes seen suggest that there is an additional role for the outer membrane fission machinery in mitochondrial distribution. One possibility is that the dendritic transport of mitochondria is functionally linked to the outer membrane dynamics. Consistent with this, TRAK1, a motor adaptor that connects mitochondria to microtubule motors, physically associates with MFN1 and MFN2 (Lee et al., 2018). Further investigation will be required to clarify how mitochondrial dynamics and trafficking are coordinated. By contrast, it has been reported that mitochondrial fragmentation is induced by the overexpression of SLC25A46 in mammalian cells and zebrafish (Abrams et al., 2015). Consistent with these observations, we found that overexpression of SLC-25A46 using the *osm-6* promoter in a wild-type background induced mitochondrial fragmentation (Fig. 5). These phenomena appear to indicate that SLC-25A46 promotes mitochondrial fission. By contrast, we have found that the phenotypes of *slc-25A46* mutants cannot be rescued by the same extrachromosomal array (Fig. 5). Instead, *slc-25A46* mutants can be rescued through the expression of *slc-25A46* by the weaker *srg-13 promoter* (Figs 1 and 5). These data show that proper expression levels of SLC-25A46 are essential for the maintenance of mitochondrial morphology, rather than indicate SLC-25A46 is a factor essential for the mitochondrial fission.

### SLC-25A46 regulates mitochondrial fusion through FZO-1

The functional relation between SLC25A46 and mitofusins has been elusive, although SLC25A46 directly binds to MFN1 and MFN2 (Janer et al., 2016). Taking advantage of worm genetics, we show that mitochondrial fusion mediated by *slc-25A46* is accomplished through the function of FZO-1, a worm MFN. The phenotype of *slc-25A46*; *fzo-1* double mutants are similar to those of single mutants, suggesting that SLC-25A46 and FZO-1 work in the same pathway in the mitochondrial fusion process. Moreover, overexpression of FZO-1 could partially suppress the phenotype of *slc-25A46*, indicating that SLC-25A46 is genetically upstream of FZO-1. It has been shown that FZO-1 is the GTPase that directly induces mitochondrial membrane fusion (Westermann, 2008). Our genetic data suggests that SLC-25A46 helps the activity of FZO-1 in the mitochondrial fusion process. It would be interesting to biochemically test whether SLC-25A46 can enhance the GTPase activity of FZO-1.

### Worm models for mitochondria-associated disorders

The mitochondrial phenotypes in model worms suggest that disease mutations induce weak loss-of-function effects on the function of SLC-25A46. This is consistent with the genotype of human individuals with pathogenic *SLC25A46* variants; most pathogenic SLC25A46 variants cause neurological disorders in an autosomal recessive manner (Abrams et al., 2015). In addition to mitochondrial defects, we found acceleration of neurodegeneration in the PVD neuron, which is a widely used model to observe neurodegeneration in *C. elegans*. Previous studies have shown that mitochondrial defects induce neurodegeneration phenotypes in worm models (Ding et al., 2022; Rawson et al., 2014). It has been shown that PVD neurons show morphological defects in the *mtx-1* mutant worm in which axonal transport of mitochondria is reduced (Zhao et al., 2021). Because the PVD neuron is the largest neuron in *C. elegans* and has more-complex dendrite structures, PVD neurons will require a lot of ATP to maintain their morphology. Neurodegeneration has been described in human individuals who have variations in the *SLC25A46* gene. Many neuronal types, both motor and sensory neurons, are affected in such individuals. We suggest that our worm models are useful to understand the connection between mitochondrial dynamics and neurodegeneration.

### Limitation of this study

Although worm genetics suggests that SLC-25A46 is a mitochondrial fusion factor, we cannot exclude a possibility that worm SLC-25A46 and mammalian SLC25A46 have different functions in the mitochondrial morphogenesis. To show that the relation between SLC-25A46 and FZO-1 is conserved in mammalian cells, experiments similar to those done in this study, such as rescue of SLC25A46-knockout cells upon the expression of MFN1 and MFN2 or analyzing of SLC25A46 and DRP1 double-knockout cells, need to be repeated in mammalian systems. Moreover, biochemical assays are required to directly show the relationship between SLC-25A46 and FZO-1.

## MATERIALS AND METHODS

### Plasmid preparation

Plasmids used in this study are detailed in Table S1. Restriction enzymes were purchased from New England Biolabs (Ipswich, MA, USA). Plasmids encoding *odr-1p::gfp* and *tomm-20(1-54aa)::gfp* were obtained from the Kang Shen laboratory (Department of Biology, Stanford University, USA). The *flp-15* promoter (*flp-15p*) and *srg-13* promoter (*srg-13p*), PHA neuron-specific promoters, were previously described (Niwa, 2016). The DNA fragment encoding *flp-15p* was inserted between the SphI and AscI restriction enzyme sites of the *tomm-20(1-54)::gfp* plasmid. The plasmid encoding *srg-13p::slc-25A46::(GGGGS)3::mCherry* was constructed based on the plasmid encoding *flp-15p::tomm-20(1-54)::gfp*. The region encoding *tomm-20(1-54)::gfp* was replaced with *slc-25A46::(GGGGS)3::mCherry,* and then the promoter region was replaced*.* Total worm cDNA from the N2 strain was prepared as previously described (Higashida and Niwa, 2023). PCR primers are listed in Table S2. *slc-25A46* cDNA was amplified by PCR using KOD FX neo DNA polymerase (TOYOBO, Tokyo Japan) with worm cDNA as the template. The slc-25A46_F_NheI and slc-25A46_R_KpnI were employed. The resulting fragment was replaced with *tomm-20(1-54)* using NheI and KpnI enzymes. Subsequently, the *gfp* sequence was replaced with *(GGGGS)3::mCherry* using Gibson assembly. *(GGGGS)3* was inserted as a flexible linker sequence (Trinh et al., 2004). To generate the plasmid encoding *srg-13p::fzo-1::(GGGGS)3::mCherry,* the *fzo-1* sequence was amplified from extracted N2 genome DNA by PCR using KOD Plus high-fidelity DNA polymerase (TOYOBO, Tokyo Japan). The region encoding *slc-25A46* was replaced with the *fzo-1* sequence.

### Worm experiments

Worm strains are described in Table S3. All *C. elegans* strains were cultured in standard nematode growth medium (NGM) plates seeded with OP50 *Escherichia coli* at 20°C (Brenner, 1974). The wild-type strain, N2 Bristol, and SD1347 carrying ccIs4251, were obtained from the *C. elegans* Genetics Center (Minneapolis, MN, USA). The deletion mutant *fzo-1(tm1133*), *eat-3(tm1107)*, and *drp-1(tm1108)* were obtained from National Bioresource Project of Japan (Tokyo Women's Medical University School of Medicine, Japan). The PVD neuron marker *wyIs592* [*ser-2prom3p::myr-gfp*] was previously described (Dong et al., 2013). For transformation of worms, DNA injection was performed as previously described (Mello et al., 1991). Plasmids encoding *flp-15p::tomm-20(1-54aa)::gfp* and *odr-1p::gfp* were injected to establish *jpnEx15*[*flp-15p::tomm-20(1-54aa)::gfp*, *flp-15p::myrTagRFP-T, odr-1p::gfp*]. The extrachromosomal array was integrated to the genome by UV irradiation. The resultant integration was named *jpnIs4*. For *slc-25A46::mCherry* and *fzo-1::mCherry* transgenic worms, 20 ng/µl of plasmids were injected into *jpnIs4* gonads, respectively. pCFJ90 (2.5 ng/µl) and pBlueScript II KS(−) (80 ng/µl) were used as co-injection markers. Then, worms carrying the extrachromosomal array were crossed to each mitochondrial mutant.

### EMS mutagenesis screening

EMS mutagenesis was performed as described previously (Brenner, 1974; Niwa, 2016). The worm carrying *jpnIs4* was crossed with N2 eight times and used as a starting strain. For suppressor screens, *slc-25A46(jpn15); jpnIs4* was used as a starting strain. Synchronized L4 stage hermaphrodites were mutagenized with 0.1 M ethyl methanesulfonate (EMS, #M0880-1G, Sigma-Aldrich). Mutagenized worms were picked onto fresh plates and left to lay eggs. F2 worms were screened under a fluorescence microscopy, and candidates were identified by the abnormal mitochondrial morphology in and abnormal localization of PHA neurons. The candidates were backcrossed to the parent line three times. The mutant locus was determined by single-nucleotide polymorphism (SNP) mapping (Davis et al., 2005). For *jpn15*, whole-genome sequencing was outsourced to Eurofins genomics Japan (Tokyo, Japan). Genomic data was analyzed using the Galaxy platform (https://usegalaxy.org/) as described previously (Community, 2022; Higashida and Niwa, 2023). Reference genome sequence (WBcel235.75.fasta) and gene annotations (WBcel235.75.gtf) were obtained from WormBase (https://wormbase.org/). All of the following sequence analysis were performed using default parameters predefined in the Galaxy platform. On the Galaxy platform, a pair of FASTQ files obtained from Eurofins were firstly trimmed with Trim Galore! (https://github.com/FelixKrueger/TrimGalore). Aligning sequencing reads to the genome sequence was performed using Bowtie 2 (https://bowtie-bio.sourceforge.net/bowtie2/index.shtml). Variant search was performed using FreeBayes (https://github.com/freebayes/freebayes). Variants were annotated to genes using Snpeff (https://pcingola.github.io/SnpEff/). By comparing the variant data and the SNP mapping data, we found a nonsense mutation in *slc-25A46* gene. The mutation was confirmed by genomic PCR followed by Sanger sequencing using CEQ8000 (Beckman Coulter, Brea, CA, USA).

For *jpn73*, SNP mapping indicated that the genomic region, which includes *drp-1*, contained the causative mutation. Therefore, the genomic region of the *drp-1* gene was amplified by PCR from *jpn73* mutant using *drp-1* genome PCR_F (5′-GGCGTTCACAGTCAATCGAAGG-3′) and drp-1 genome PCR_R (5′-GGGAACGGAGCATAGAGATCATACAG-3′) primers. The genomic DNA fragment was separated by electrophoresis using a 1% agarose gel and purified using QIAquick Gel Extraction Kit (QIAGEN). The genome sequence of *drp-1* was determined by Sanger sequencing using primers listed in Table S2.

### Genome editing

The co-CRISPR method was used for genome editing of worms (Arribere et al., 2014). The single-strand oligonucleotide DNA (ssODN) with *dpy-10 (cn64)* was used as a co-injection marker. Target sequences for guide RNA were inserted to an expression vector pTK73 (Obinata et al., 2018). We chose two Cas9 target cites in *slc-25A46* sequence for each experiment. To correct the *jpn15* mutation, target sites were: *jpn15*_rescue_01, 5′-GAATTCAGACATTCTAGAA-3′ and *jpn15*_rescue_02, 5′-CATTCTAGAAAGGAGCAAT-3′. To introduce disease associated mutations, target sites were: *slc-25A46*_disease_01, 5′-GATGAACAATTGTTTCAAA-3′ and *slc-25A46*_disease_02, 5′-TTCATCGAATGTATATTCA-3′.

Synthesized ssODN (Eurofins genetics Japan) were used as repair templates (Table S3). We introduced restriction enzyme sites as synonymous mutations to guide RNA recognition cites to prohibit the cleavage of the repair templates and for following genotyping process. All materials were mixed so that the final concentration of guide RNA vectors, Cas9-expression vector and ssODN was 50 ng/µl, and then injected the mixture into worms. *dpy* or *rol* F1 worms were singled under a stereo microscope (Stemi 508, Carl Zeiss). Recombination was screened by PCR followed by digestion with each restriction enzyme, and then confirmed by Sanger sequencing. Obtained lines were crossed with N2 worms three times. All of the plasmids, primers and ssODNs are listed in Table S1 and S2.

### Fluorescence microscopy

Fluorescent signals in living worms were observed without fixation, following the previously described procedure (Anazawa and Niwa, 2022). Worms were mounted on a 3% agarose pad containing 0.25 mM levamisole (Sigma). We used a Zeiss Axio Observer microscope equipped with an Objective C-Apochromat 40x (NA 1.2) and an LSM800 confocal microscope system (Carl Zeiss). The system was controlled using ZEN software (Carl Zeiss). Airy unit settings were configured to 2, and the *Z*-stack mode was employed to capture images. Parameters for resolution in the *X-Y* direction and thickness in the *Z*-direction were the optimal values indicated by the ZEN software. *Z*-projection was performed using the same software.

### Measurement of mitochondrial size and number

To quantify the mitochondrial size, individual mitochondria were manually selected using Freehand selections tool in FIJI (ImageJ), and size measurement were obtained using the Measure function. Because mitochondria within dendrites have a thickness in the *Z*-direction less than the wavelength of light, mitochondrial size was expressed as an area on the *Z*-projection image rather than volume in this study. In PHA neurons, the number of mitochondria was counted in each cell by visual inspection of fluorescence images. In PVD neurons, the number of mitochondria was counted in a 100 µm segment of the primary dendrite extending from the cell body. For categorization of mitochondrial phenotypes in Figs 3C and 6D, mitochondrial morphology was visually classified into four categories shown in Fig. 3B, based on fluorescent images. ‘Weakly fragmented’ mitochondria were defined as consisting of predominantly short tubules with occasional discontinuities, whereas ‘highly fragmented’ mitochondria appeared as numerous small, discrete puncta totally lacking tubular continuity. When each mitochondrial dots were not clear, the phenotype was classified as ‘aggregated’. Classifications were made by visual inspection. The researchers doing this analysis were aware of the experimental details.

### Rapid cooling procedure and quick freeze substitution for electron microscopy

The cryofixation of worms was conducted using the ultra-rapid cryo-technique as described previously (Irdani et al., 2015). Briefly, adult animals were picked onto four NGM plates (8–10 animals/plate) with OP50 bacteria and cultured at 20°C. On the third day of culture, a mixture of L1–L4 animals was collected using a cryoprotective solution [10% ethylene glycol in deionized distilled water (DDW)] and incubated for more than 30 min at room temperature. The drop of animals was loaded onto a small cellulose acetate membrane (Membrane filter C020A047A, ADVANTEC). After the liquid volume reduction by absorption into the membrane, the samples were dipped manually into liquid ethane or liquid propane and then transferred into liquid nitrogen. The samples were further transferred to cryotubes (Cryovial, 2 ml, TOHO) containing 2% OsO_4_ and 5% DDW in acetone at −80°C. The specimens were freeze substituted by the quick freeze substitution (QFS) method (McDonald and Webb, 2011), washed five times with acetone and three times with propylene oxide (PO) and infiltrated with Epon resin/PO in steps of 33% 1 h, 50% 3 h and 100% resin overnight. After infiltration, the specimens were embedded in freshly prepared Epon resin and polymerized for 2 days at 60°C.

### Electron microscopy

70 nm ultrathin sections were prepared onto Formvar-coated copper slot grids using an ultramicrotome (EM UC7, Leica). The sections were post-stained for 5 min in 5% uranyl acetate in water at 60°C and followed by 5 min in lead citrate. Mitochondria in body-wall muscles were photographed using a JEM-2100F electron microscope (JEOL) equipped with TemCam-F216 (TVIPS) at 200 kV and 10,000×.

### Statistical analyses and graph preparation

Statistical analyses were performed using Graph Pad Prism version 10.1.1. Statistical methods and sample size are described in the figure legends. Prior to selecting statistical tests, the distribution of each dataset was assessed by visual inspection of dot plots. As the data deviated from normality in Figs 1–6, nonparametric tests were used. Median values are shown on these graphs. In Fig. 7, parametric tests were applied. Graphs were prepared using Graph Pad Prism version 10.1.1, exported in the EPS file format and aligned by Adobe Illustrator 2023.

### AI tool use statement

During the preparation of this work the authors used ChatGPT in order to check English grammar and improve English writing. After using this tool, the authors reviewed and edited the content as needed and take full responsibility for the content of the publication.

## Supplementary Material



10.1242/joces.263571_sup1 Supplementary information
