# Supplementary material for: SLC-25A46 regulates mitochondrial fusion through the mitofusin protein FZO-1 and is essential for maintaining neuronal morphology
Source: J Cell Sci. 2025 Jun 23;138(12):jcs263571. doi: 10.1242/jcs.263571 (PMC12273626; doi:10.1242/jcs.263571)
Supplement: Supplementary information [file joces-138-263571-s1.pdf]

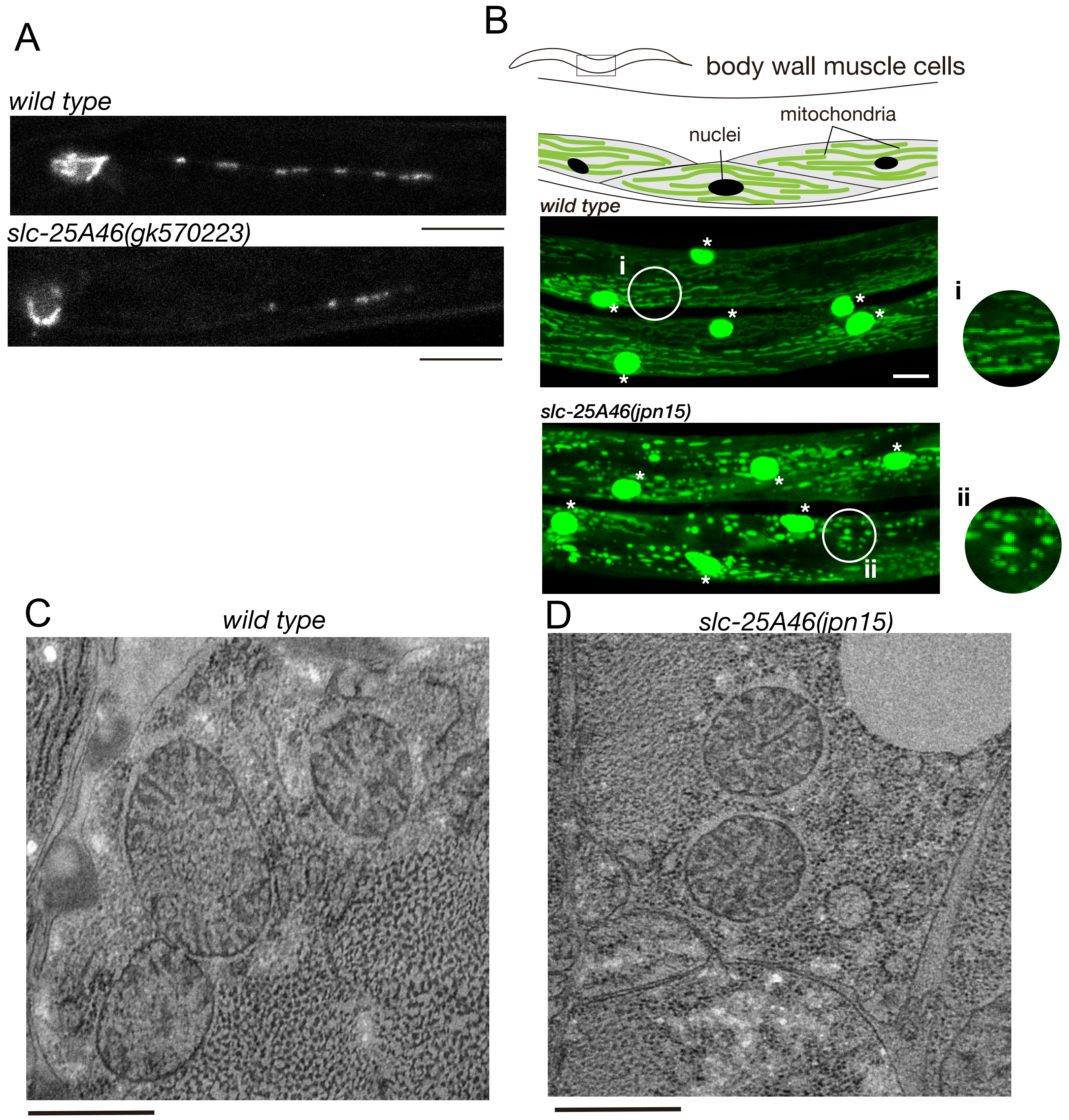

Fig. S1.

(A) Representative images showing the morphology and distribution of mitochondria in PHA neuron of wild type and slc-25A46(gk570223). Bars, 10 μm.

(B) Representative fluorescent microscopic images showing the morphology of mitochondria in muscular cells of wild type and slc-25A46(jpn15). Bars, 10 μm.

(C and D) Representative transmission electron microscopic images showing the morphology of mitochondria in muscular cells of wild type and slc-25A46(jpn15). Bars, 100 nm.

Journal of Cell Science • Supplementary information

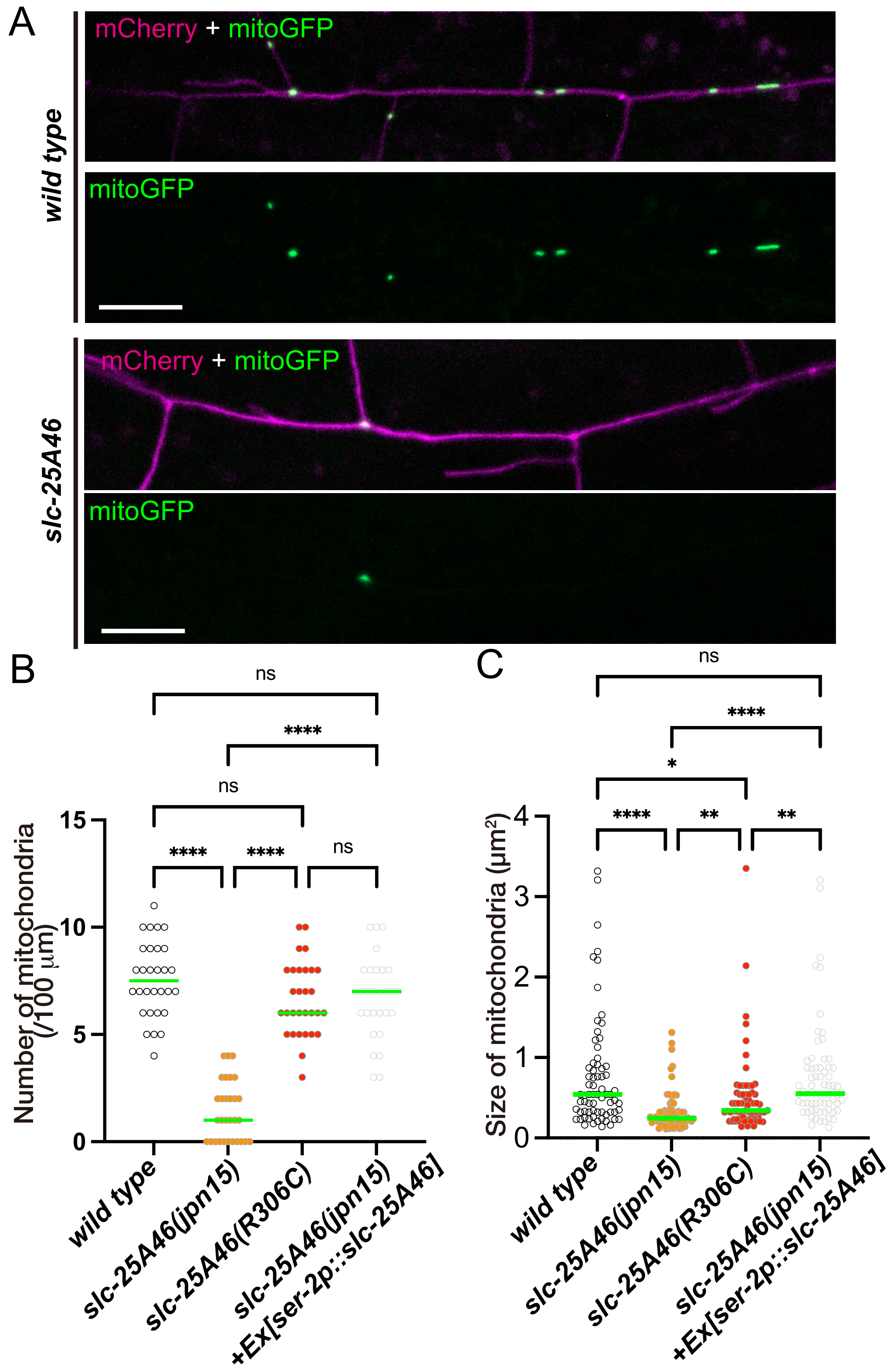

Fig. S2.

(A) Representative images showing the mitochondrial morphology in the PVD neuron of wild type and slc-25A46(jpn15) mutants. Mitochondria were visualized using TOMM-20::GFP (green), and mCherry (magenta) was expressed to visualize cell morphology. Upper panels show merged images. Lower panels show mitochondria alone.

(B) Dot plots showing the number of mitochondria within a 100 μm segment of the PVD dendrite. Each dot represents the number of mitochondria in a single segment. Green bars represent median values.

n = 30 dendrites from 30 animals per genotype. Kruskal-Wallis test followed by Dunn' s multiple comparisons test. ****, $p < 0.0001$. ns, $p > 0.05$ and statistically not significant.

(C) Dot plots showing the size distribution of mitochondria in PVD dendrites. Each dot represents the size of an individual mitochondrion. Green bars indicate median values. n = 70 mitochondria for each genotype. Kruskal-Wallis test followed by Dunn' s multiple comparisons test. *, $p < 0.05$. ***, $p < 0.001$. ****, $p < 0.0001$.

Table S1. Plasmid list

plasmid name	insert	description
pSM	worm GFP vector	from Kang Shen lab (Stanford Univ.)
ΔpSM	empty vector for worm expression	from Kang Shen lab (Stanford Univ.)
Podr-1::gfp	*odr-1p::gfp*	from Kang Shen lab (Stanford Univ.)
tomm-20(1-54aa)::gfp	*tomm-20(1-54aa)::gfp*	from Kang Shen lab (Stanford Univ.)
Pflp-15::gfp	*flp-15p::gfp*	Niwa(2015) Scientific reports
pSN290	*flp-15p::tomm-20(1-54aa)::gfp*	This study
pSN291	*flp-15p::myrTagRFP-T*	This study
pCFJ90	*myo-2p::mCherry::unc-54_3'UTR*	from Addgene
pOB28	*flp-15p::slc-25A46(cDNA)::(GGGGS)3::mCherry::unc-54_3'UTR*	This study
pSN1092	*srg-13p::slc-25A46(cDNA)::(GGGGS)3::mCherry::unc-54_3'UTR*	This study
pSN1093	*srg-13p::slc-25A46(cDNA)::unc-54_3'UTR*	This study
pSN331	*osm-6p::slc-25A46(cDNA)::unc-54_3'UTR*	This study
pSN1091	*ser-2prom3::slc-25A46(cDNA)::unc-54_3'UTR*	This study
pSN1090	*srg-13p::fzo-1::mCherry::unc-54_3'UTR*	This study
pTK73	*CeU6 promoter ::sgRNA (F+E) scaffold sequence*	This study
pTK73_rescue-1	*CeU6 promoter :: target sequence (GAATTCAGACATTCTAGAA) :: sgRNA (F+E) scaffold sequence*	This study
pTK73_rescue-2	*CeU6 promoter :: target sequence (CATTCTAGAAAGGAGCAAT) :: sgRNA (F+E) scaffold sequence*	This study
pTK73_P299L&E301D&R306C-1	*CeU6 promoter :: target sequence (GATGAACAATTGTTTCAAA) :: sgRNA (F+E) scaffold sequence*	This study
pTK73_P299L&E301D&R306C-2	*CeU6 promoter :: target sequence (TTCATCGAATGTATATTCA) :: sgRNA (F+E) scaffold sequence*	This study

Journal of Cell Science • Supplementary information

Table S2.Oligonucleotide list

oligo name	sequence	comment
**slc-25A46_F_NheI**	atGCTAGC ATGCCTACACAATTCATTAGGAACCGG	cloning of slc-25A46
**slc-25A46_R_KpnI**	atgc GGTACC cc ACCCGAAAATGGGTCTCCAGACGAC	cloning of slc-25A46
**drp-1 genome PCR_F**	GGCGTTCACAGTCAATCGAAGG	sequencing of drp-1
**drp-1 genome PCR_R**	GGGAACGGAGCATAGAGATCATACAG	sequencing of drp-1
**jpn73_seq_primer_exon1**	TTCGCACGGCATCGAAGTCTGG	sequencing of drp-1
**drp-1exon2-1_seqF**	TACTGGCTCTAAGGTTTTCACAG	sequencing of drp-1
**drp-1exon2-2_seqF**	CAGGATTTCGCTACTTCGGAGCC	sequencing of drp-1
**drp-1exon3-1_seq**	TATTTGGCAAAGAGATTGAATATGG	sequencing of drp-1
**drp-1exon3-2_seq**	AATGCAACGAATGGTTCAGCATTGC	sequencing of drp-1
**drp-1exon4_seq**	tgccaggaagtgcggatgactg	sequencing of drp-1
**drp-1exon5&6_seq**	ATGTCGCTATTATCGgtatgagacc	sequencing of drp-1
**jpn15jpn33_PCR_F**	CTGAGCCTCACCCAATCTCGAAATC	sequencing of jpn15 and jpn33
**jpn15jpn33_PCR_R**	ATTCGATCCACACCCTTCACAAGAAC	sequencing of jpn15 and jpn33
**jpn15jpn33_seq_primer**	TCGCTAATTTCCCATCCATGCGGTG	sequencing of jpn15 and jpn33
**dpy-10(cn64)_oligo**	TGAAGCCATGTGAAGCTCCGCTACCATAGGCACCACAAGCGGTACGG GTTCCAGTCATTCTCATCTTGCCGTATTGAAGTTCAAGTGCAGCCTCG TCGTTTGATCTC	repair template for dpy-10
**P299L_ssODN**	ATCATCAATGGTGTTAACTGATTTGATACTTTATCTTTTCGAGACCATC GTGCACAGAATGTACATCCAAGGAACACGAACACTTATTGATAATAT GGAT	repair template for disease mutation
**E301D_ssODN**	CATCAATGGTGTTAACTGATTTGATACTTTATCCATTCGATACCATCGT GCACAGAATGTACATCCAAGGAACACGAACACTTATTGATAATATGGATAC	repair template for disease mutation
**slc_R306C_ssODN**	ATCAATGGTGTTAACTGATTTGATACTTTATCCATTCGAGACCATCGTGC ACTGCATGTACATCCAAGGAACACGAACACTTATTGATAATATGGATACA	repair template for disease mutation
**jpn15rescue_ssODN**	CTGATTCCTGTAATCTGTAATTCTGTCGCTAAACAAGGAATCCAAACC TTTTGGAAGGGCGCCATCGGCTCAAGTGTGCTCTGGGGCCTCACGAA TGTTACGGAA	introducing jpn33 mutation in jpn15

Journal of Cell Science • Supplementary information

J. Cell Sci.: doi:10.1242/jcs.263571: Supplementary information

Table S3. Strain list

strain name	genotype	source and commments
N2	*wild type*	from CGC
OTL45	*jpnEx15[flp-15p::mito::GFP; flp-15p::myrTagRFP-T; odr-1p::GFP]*	this study, mitochondria is visualized with GFP in the PHA neuron
OTL48	*jpnIs4[flp-15p::mito::GFP; flp-15p::myrTagRFP-T; odr-1p::GFP] V*	this study, mitochondria is visualized with GFP in the PHA neuron
OTL247	*slc-25A46(jpn15)I; jpnIs4V*	this study, null mutant of slc-25A46 obtained through EMS mutagenesis, Figure 1 - 6.
OTL249	*slc-25A46(jpn33)I; jpnIs4V*	this study, *jpn15* mutation was corrected by CRISPR/Cas9, Figure 1
VC40317	*slc-25A46(gk570223)*	from CGC
OTL277	*slc-25A46(gk570223); jpnIs4V*	this study, Figure S1
SD1347	*ccIs4251*	from CGC, Figure S1
OTL260	*slc-25A46(jpn15);ccIs4251*	this study, Figure S1
	fzo-1(tm1133)II	from NBRP
	eat-3(tm1107)	from NBRP
	drp-1(tm1108)	from NBRP
OTL252	*drp-1(tm1108)IV; jpnIs4V*	this study, Figure 2 and 3
OTL241	*slc-25A46(jpn15); drp-1(jpn73); jpnIs4*	this study, obtained through EMS mutagenesis, Figure 2
OTL242	*drp-1(jpn73); jpnIs4V*	this study, Figure 2
OTL251	*fzo-1(tm1133)II; jpnIs4V*	this study, Figure 3 and 4
OTL250	*eat-3(tm1107); jpnIs4V*	this study, Figure 3
OTL93	*slc-25A46(jpn15)I; fzo-1(tm1133)II; jpnIs4V*	this study, Figure 4
OTL327	*jpnIs4V; jpnEx585 [srg-13p::fzo-1, odr-1p::RFP]*	this study, Figure 4
OTL329	*slc-25A46(jpn15)I; jpnIs4V; jpnEx585 [srg-13p::fzo-1, odr-1p::RFP]*	this study, Figure 4
OTL332	*fzo-1(tm1133)IV; jpnIs4V; jpnEx585 [srg-13p::fzo-1, odr-1p::RFP]*	this study, Figure 4
OTL328	*jpnIs4V; jpnEx586 [srg-13p::slc-25A46, odr-1p::RFP]*	this study, Figure 4 and 5
OTL330	*slc-25A46(jpn15); jpnIs4V; jpnEx586 [srg-13p::slc-25A46, odr-1p::RFP]*	this study, Figure 4
OTL333	*fzo-1(tm1133); jpnIs4V; jpnEx586 [srg-13p::slc-25A46, odr-1p::RFP]*	this study, Figure 4
OTL336	*jpnEx588[srg-13p::gfp; odr-1p::rfp]*	for quantification of promoter strength
OTL337	*jpnEx589[srg-13p::gfp, odr-1p::rfp]*	for quantification of promoter strength
OTL338	*jpnEx590[osm-6p::gfp, odr-1p::rfp]*	for quantification of promoter strength
OTL339	*jpnEx591[osm-6p::gfp, odr-1p::rfp]*	for quantification of promoter strength
OTL270	*jpnIs4 ; jpnEx53[Posm-6::slc-25A46,myo-2p::RFP]*	this study, Figure 5
OTL253	*slc-25A46(jpn37); jpnIs4V*	this study, P299L mutation in the slc-25A46 gene, Figure 6
OTL254	*slc-25A46(jpn38); jpnIs4V*	this study, E301D mutation in the slc-25A46 gene, Figure 6
OTL255	*slc-25A46(jpn39); jpnIs4V*	this study, R306C mutation in the slc-25A46 gene, Figure 6
TV15911	*wyIs592[ser-2prom-3p::myr-GFP, odr-1p::rfp]*	from Kang Shen lab(Stanford Univ.), GFP is expressed in the PVD neuron, Figure 7
OTL259	*slc-25A46(jpn15); wyIs592*	this study, Figure 7
OTL256	*slc-25A46(jpn37); wyIs592*	this study, P299L mutation in the slc-25A46 gene, Figure 7
OTL257	*slc-25A46(jpn38); wyIs592*	this study, E301D mutation in the slc-25A46 gene, FIgure 7
OTL58	*slc-25A46(jpn39); wyIs592*	this study, R306C mutation in the slc-25A46 gene, Figure 7
TV51447	*wyIs50054[ser-2prom3::tomm-20(1-54)::gfp, ser-2prom3::myr-mcherry, odr-1p::gfp]*	from Kang Shen lab(Stanford Univ.), Figure S2
OTL326	*slc-25A46(jpn15); jpnIs4; wyIs50054*	this study, Figure S2
OTL334	*slc-25A46(jpn39); jpnIs4; wyIs50054*	this study, Figure S2
OTL335	*slc-25A46(jpn15); jpnIs4; wyIs50054; jpnEx587[ser-2prom3::slc-25A46; myo-2p::RFP]*	this study, Figure S2

Journal of Cell Science • Supplementary information
