# Supplementary material for: SLC-25A46 regulates mitochondrial fusion through the mitofusin protein FZO-1 and is essential for maintaining neuronal morphology
Source: J Cell Sci. 2025 Jun 23;138(12):jcs263571. doi: 10.1242/jcs.263571 (PMC12273626; doi:10.1242/jcs.263571)
Supplement: Supplementary file 1 [file joces-138-263571_review_history.pdf]

SLC-25A46 regulates mitochondrial fusion through the mitofusin protein FZO-1 and is essential for maintaining neuronal morphology

Hiroyuki Obinata, Taisei Watanabe, Hironori Takahashi, Satoshi Shimo, Toshiyuki Oda, Asako Sugimoto and Shinsuke Niwa
DOI: 10.1242/jcs.263571

Editor: Heidi M McBride

Review timeline
Original submission: 24 September 2024
Editorial decision: 11 November 2024
First revision received: 23 April 2025
Accepted: 22 May 2025

Original submission

First decision letter

MS ID#: jcs.263571

MS TITLE: SLC-25A46 Regulates Mitochondrial Fusion through FZO-1/Mitofusin and is Essential for Maintaining Neuronal Morphology

AUTHORS: Hiroyuki Obinata; Hironori Takahashi; Satoshi Shimo; Toshiyuki Oda; Asako Sugimoto; Shinsuke Niwa

ARTICLE TYPE: Research Article

Dear Dr Niwa,

We have now reached a decision on the above manuscript.

To see the reviewers' reports and a copy of this decision letter, please go to:

As you will see, the reviewers raise a number of substantial criticisms that prevent me from accepting the paper at this stage. Both reviewers had significant concerns that would need to be addressed, particularly given the existing controversies over the function of SLC25A46 in the literature. The use of genetic models is certainly a strength that was recognized by the reviewers. They suggest, however, that a revised version might prove acceptable, if you can address their concerns. If you think that you can deal satisfactorily with the criticisms on revision, I would be pleased to see a revised manuscript. We would then return it to the reviewers.

Reviewer 1

Advance summary and potential significance to field

Summary:
In this study, Obinata et al. investigate the genetic relationships between SLC-25A46, an outer mitochondrial membrane protein in the solute carrier protein family, and known components of the mitochondrial fission and fusion machinery. Previous studies have identified recessive mutations in

© 2025. Published by The Company of Biologists under the terms of the Creative Commons Attribution License (https://creativecommons.org/licenses/by/4.0/).

SLC25A46 that cause dominant optic atrophy and axonal peripheral neuropathy in humans (Abrams et al. 2015), and several studies have reported that mutations in SLC25A46 lead to disrupted mitochondrial morphology in various model systems. However, previous studies have reported conflicting effects of SLC25A46 mutations: some have reported excessive mitochondrial fusion, suggesting that SLC25A46 is a mitochondrial fission factor, while others have observed mitochondrial fragmentation, suggesting that SLC25A46 is involved in mitochondrial fusion. In this study, the authors employ unbiased forward genetic screening in C. elegans and identify slc-25A46 as a gene involved in mitochondrion morphology. In slc-25A46(jpn15) mutant worms harboring a nonsense mutation, the number and size of mitochondria in a sensory dendrite are decreased. Subsequent genetic epistasis experiments demonstrated that slc-25A46 genetically acts upstream of mitochondrial fusion machinery, suggesting that SLC-25A46 plays a role in mitochondrial fusion.

Merits of this study:
Previous studies have reported conflicting effects of SLC25A46 mutations on mitochondrial morphology. The present study offers relevant genetic evidence that SLC-25A46 acts to enable or promote mitochondrial fusion. It is my opinion that the most valuable and novel contributions of this study to the field are the following: 1) A nonsense allele of slc-25A46 leads to mitochondrial fragmentation in vivo, which phenocopies the effects of fzo-1 mutations, 2) loss-of-function mutations in the fission factor drp-1 suppress this fragmentation, 3) double mutants of slc-25A46 and fzo-1 do not exhibit stronger fragmentation phenotypes than fzo-1 alone, arguing that both genes act in the same genetic pathway, and 4) overexpression of FZO-1 can overcome the fragmentation phenotype of slc-25A46 mutants. These results offer important clues for understanding the cell biological mechanisms of mitochondrial morphology deficits in slc-25A46 mutant backgrounds.

Limitations of this study:
Beyond the aforementioned genetic epistasis experiments placing slc-25A46 genetically upstream of the mitochondrial fusion machinery, the present study offers little insight into the cell biological mechanisms of how SLC-25A46 acts to promote mitochondrial fusion. While valuable, these genetic experiments are not sufficient to stand on their own for publication. The authors do also report the effects of human missense mutations on mitochondrial fragmentation and neuronal morphology. However, the missense mutations reported in Figure 5 are observational in nature and offer little additional insight beyond replicating the known effects of these mutations in other systems; furthermore, the analysis of neuronal morphology in Figure 6 reports a well-known phenomenon of axon and dendrite degeneration in mutant backgrounds that disrupt mitochondrial transport, positioning, and morphology (for example, see Rawson et al. 2014, Zhao et al. 2021, and Ding et al. 2022).

Comments for the author

Major Points:
1) The wildtype control data for mitochondria number are re-used in Figures 1C, 3F, 4B, and 4E; however, this fact is not acknowledged in the figure legends and is thus inappropriately misleading to the reader. The authors must do separate control experiments for each figure.

2) It is critical that the authors determine which cells SLC-25A46 functions in. Currently, cell-specific expression of SLC-25A46 did not rescue the phenotype, raising concerns about whether the phenotypes are indeed caused by a cell autonomous function. In addition, the localization of SLC-25A46 to mitochondria must be demonstrated, otherwise the authors cannot rule out an indirect effect.

3) The present study offers little insight into the cell biological mechanisms of how SLC-25A46 acts to promote mitochondrial fusion. The authors should make a prediction based on their data and test it experimentally. For example, what is the localization of FZO-1 in slc-25A46 mutant animals compared to wildtype? Does FZO-1 colocalize with SLC-25A46?

Minor Revisions:
4) The figures are incomplete and lack sufficient detail. In particular, the following areas must be addressed for completeness and clarity:

© 2025. Published by The Company of Biologists under the terms of the Creative Commons Attribution License (https://creativecommons.org/licenses/by/4.0/).

* Figure 2 has no quantifications and is unacceptable for publication in its current form. These phenotypes should be quantified. Furthermore, the dendrite of the slc-25A46; drp-1 double mutant needs to be shown in addition to the cell body. Please quantify and report the degree of rescue in mitochondrial number and size.
* For the TEM in Figure S1, please adjust the brightness and contrast settings so that the images are not so dark. These images are not informative without quantifications. Please provide a quantification of these data (this could be size, shape, circularity, circumference, or number of cristae).
* Figure 4 E and F lack labels on the Y axis.
* Figure 4 E and F are very confusing to the reader. There is no way to know from the figure, or from the figure legend, that "Ex[slc-25a46]" refers to a fosmid with the slc-25a46 genomic DNA. (Figure S2 uses the same "Ex[slc-25a46]" nomenclature to refer to cDNA driven by the flp-15 promoter.) The only way to distinguish the two is by referencing the text. At first glance, Figures 4 and S2 appear to completely contradict each other. Please add labels to 4 E and F, as well as the figure legend, to clarify the use of a fosmid in this panel. Labels should be added to Figure S2 and its legend as well.
* In Figure 4 E and F, please show the statistical significance between the array(-) and array(+) strains within the slc-25a46 and fzo-1 backgrounds.

5) The lack of phenotype enhancement in the slc-25a46; fzo-1 double mutant is one of the strongest pieces of evidence substantiating the claim that SLC-25A46 acts upstream of FZO-1. It is my opinion that this result should be reported in the main figure rather than in the supplement.

6) The Methods section lacks sufficient detail. In particular, the following areas need to be addressed:
* How was mitochondrion size / area measured and quantified?
* What criteria were used to define and score fragmentation categories? This is especially important for the reader to evaluate the value of the data presented in Figure 5.
* How was "branch occupancy" measured in Figure 6, and what does this metric mean?

7) The text needs to be revised for completeness. The following areas should be addressed:
* Figure 1 C and D should be explicitly discussed in the text.

8) The title contains the phrase, "and is Essential for Maintaining Neuronal Morphology." While this phrase may be technically accurate from a genetics standpoint, this property is certainly not unique to SLC-25A46, and could be said of any number of proteins involved in mitochondrial transport, positioning and morphology. Furthermore, the role of SLC-25A46 is almost certainly quite a few steps upstream of neurodegeneration and neuronal morphology. It is my opinion that this phrase unnecessarily exaggerates the importance of the findings and should be removed from the title. In addition, since neuronal integrity was examined in a neuron in which the authors did not examine mitochondrial phenotypes, this conclusion is a bit misleading.

9) The authors report defects in the morphology of the PVD neuron in slc-25A46 mutants. I would suggest removing this figure unless mitochondria are also examined in this neuron.

10) It is unclear why nonparametric statistical tests were used exclusively throughout the paper. Were the data non-normally distributed? The data points visually do not appear to be so. The rationale for this choice should be explained in the Methods; otherwise, standard one-way ANOVAs with the mean and SD error bars should be plotted rather than the median.

<u>Reviewer 2</u>

Advance summary and potential significance to field

Through unbiased forward genetic screening in C. elegans , Obinata et al discovered that SLC-25A46 is essential for maintaining mitochondrial and neuronal morphology. Their findings revealed that SLC-25A46 functions upstream of fzo-1 in regulating mitochondrial morphology. They also explored the impact of disease-related mutations in SLC-25A46 on mitochondrial and neuronal morphologies. Notably, their findings suggested that the expression level of SLC-25A46 influences mitochondrial morphology, which helps to explain inconsistencies in previous studies regarding its role in

© 2025. Published by The Company of Biologists under the terms of the Creative Commons Attribution License (https://creativecommons.org/licenses/by/4.0/).

mitochondrial fission and fusion. Overall, the manuscript is well-written, and the data derived from a well-designed experiment are sound. However, some conclusions are not fully supported by the data. The following points should be addressed before publication in JCS.

Major concerns;
1. In Figure 1A, B, the mitochondrial signal appears saturated, and the images are too small to clearly depict mitochondrial morphology. Please provide unsaturated, magnified images of mitochondria in both cell bodies and dendrites.
2. The authors observe that the loss of SLC25A46 (jpn15) leads to a decrease in both the number of mitochondria per dendrite and the size of individual mitochondria (Fig. 1C and 1D). Inhibition of mitochondrial fusion would increase the number of mitochondria, similar to the eat-3 mutant phenotype shown in Fig. 3F and 3G. How do the authors explain the reduction in the number of mitochondria in dendrites of the SLC25A46 mutant?
3. Single images from each mutant line are not sufficient to demonstrate the mitochondrial fragmentation phenotype in the cell body in Fig. 2A. As this phenotype is used as an indicator for identifying suppressor mutants of the jpn15 allele, it should be quantified and statistically analyzed.
4. The definition of "bead-like structures" is missing in Fig 6B. Clear definition of the bead-like structures used for quantification in Fig. 6C and arrows to indicate them in Fig. 6B, probably with higher magnification images, are required.
5. The authors showed that jpn15 and disease-related mutants exhibit abnormal neuronal morphology in PVD neurons. Is this phenotype accompanied by abnormal mitochondrial morphology? Mitochondria morphology should be investigated in PVD neurons.

Minor points;
1. On page 9 lines 1-2, "The mammalian SLC25A46 has been reported to physically interact with the outer and inner membrane fusion factors MFN1 and MFN2 (FZO-1 orthologs) (Steffen et al., 2017)." Does it mean MFN1 and MFN2 (or one of those) are inner membrane fusion factors as well? If not, an inner membrane fusion factor should be named here.
2. On page 9 line 17: Fig 4G instead of Fig 4D
3. On page 10 lines 9-12: It appears that Fig 5F and Fig 5G have been swapped.
4. Fig 6B: What do the asterisks in the images indicate? Please mention it in the figure legend.
5. The discussion regarding how the expression level of SLC25A46 can influence the phenotype of mitochondrial morphology is very informative. However, the statement on page 11, line 19, "an observation in mutant mice considered to be hypomorph," which should refer to [Li et al., 2017, Hum. Mol. Genet. 26, 3776-3791, PMID: 28934388], appears to describe the original paper's claim incorrectly. What is the rationale for using the term "hypomorph"? In their paper, Li et al. created a transgenic knockout mouse by inducing a 46-bp deletion in exon 8 (C-terminal) and confirmed that endogenous Slc25a46 signals were undetectable in western blot analysis using antibodies targeting the N-terminal of Slc25a46 in the Slc25a46-/- cortex and cerebellum.

First revision

<u>Author response to reviewers' comments</u>

Reviewer 1: SUMMARY OF THE ADVANCE MADE IN THIS PAPER AND ITS POTENTIAL SIGNIFICANCE TO THE FIELD
Summary:
In this study, Obinata et al. investigate the genetic relationships between SLC-25A46, an outer mitochondrial membrane protein in the solute carrier protein family, and known components of the mitochondrial fission and fusion machinery. Previous studies have identified recessive mutations in SLC25A46 that cause dominant optic atrophy and axonal peripheral neuropathy in humans (Abrams et al. 2015), and several studies have reported that mutations in SLC25A46 lead to disrupted mitochondrial morphology in various model systems. However, previous studies have reported conflicting effects of SLC25A46 mutations: some have reported excessive mitochondrial fusion, suggesting that SLC25A46 is a mitochondrial fission factor, while others have observed

© 2025. Published by The Company of Biologists under the terms of the Creative Commons Attribution License (https://creativecommons.org/licenses/by/4.0/).

mitochondrial fragmentation, suggesting that SLC25A46 is involved in mitochondrial fusion. In this study, the authors employ unbiased forward genetic screening in C. elegans and identify slc-25A46 as a gene involved in mitochondrion morphology. In slc-25A46(jpn15) mutant worms harboring a nonsense mutation, the number and size of mitochondria in a sensory dendrite are decreased. Subsequent genetic epistasis experiments demonstrated that slc-25A46 genetically acts upstream of mitochondrial fusion machinery, suggesting that SLC-25A46 plays a role in mitochondrial fusion.

Merits of this study:
Previous studies have reported conflicting effects of SLC25A46 mutations on mitochondrial morphology. The present study offers relevant genetic evidence that SLC-25A46 acts to enable or promote mitochondrial fusion. It is my opinion that the most valuable and novel contributions of this study to the field are the following: 1) A nonsense allele of slc-25A46 leads to mitochondrial fragmentation in vivo, which phenocopies the effects of fzo-1 mutations, 2) loss-of-function mutations in the fission factor drp-1 suppress this fragmentation, 3) double mutants of slc-25A46 and fzo-1 do not exhibit stronger fragmentation phenotypes than fzo-1 alone, arguing that both genes act in the same genetic pathway, and 4) overexpression of FZO-1 can overcome the fragmentation phenotype of slc-25A46 mutants. These results offer important clues for understanding the cell biological mechanisms of mitochondrial morphology deficits in slc-25A46 mutant backgrounds.

Limitations of this study:
Beyond the aforementioned genetic epistasis experiments placing slc-25A46 genetically upstream of the mitochondrial fusion machinery, the present study offers little insight into the cell biological mechanisms of how SLC-25A46 acts to promote mitochondrial fusion. While valuable, these genetic experiments are not sufficient to stand on their own for publication. The authors do also report the effects of human missense mutations on mitochondrial fragmentation and neuronal morphology. However, the missense mutations reported in Figure 5 are observational in nature and offer little additional insight beyond replicating the known effects of these mutations in other systems; furthermore, the analysis of neuronal morphology in Figure 6 reports a well-known phenomenon of axon and dendrite degeneration in mutant backgrounds that disrupt mitochondrial transport, positioning, and morphology (for example, see Rawson et al. 2014, Zhao et al. 2021, and Ding et al. 2022).
We agree with the reviewer and discussed these previous findings in the revised manuscript. Moreover, we improved the manuscript by adding new data.

SUGGESTIONS TO AUTHORS
Major Points:
1) The wildtype control data for mitochondria number are re-used in Figures 1C, 3F, 4B, and 4E; however, this fact is not acknowledged in the figure legends and is thus inappropriately misleading to the reader. The authors must do separate control experiments for each figure.
We improved the statistical analysis to avoid type I error. Thank you very much for the suggestion.

2) It is critical that the authors determine which cells SLC-25A46 functions in. Currently, cell-specific expression of SLC-25A46 did not rescue the phenotype, raising concerns about whether the phenotypes are indeed caused by a cell autonomous function. In addition, the localization of SLC-25A46 to mitochondria must be demonstrated, otherwise the authors cannot rule out an indirect effect.
When we overexpressed *slc-25A46* cDNA by using ether *flp-15* or *osm-6* promoter, mitochondria were fragmented (original manuscript and new Figure 5). Therefore, we instead tried *srg-13* promoters, which is also a PHA-neuron specific promoter at the tail region. As a result, *Psrg-13::slc-25A46* did not cause fragmentation of mitochondria in the wild type background (new Figure 5). We think this is due to the expression levels. Compared to *osm- 6* promoter, the expression level of *srg-13* promoter is much weaker as described in the revised manuscript. In average, the osm-6 promoter is 8.5 times stronger than srg-13 promoter as following:
"Previous studies have shown that overexpression of SLC25A46 induces mitochondrial fragmentation in zebrafish and mammalian cells. To test whether the phenotype was associated with differences in promoter strength, we compared the expression levels of osm-6 and srg-13 promoter by quantifying mean GFP fluorescent intensity in PHA neurons of transgenic worms carrying extrachromosomal Posm-6::gfp or Psrg-13::gfp reporter arrays

© 2025. Published by The Company of Biologists under the terms of the Creative Commons Attribution License (https://creativecommons.org/licenses/by/4.0/).

(Supplemental Table S3, OTL336-339). When the fluorescent intensity of GFP were measured in PHA neurons, the expression level of the osm-6 promoter was approximately 8.5-fold higher than that of the srg-13 promoter (mean ±S.D.: 35027 ± 5289 for Posm-6::gfp and 4159 ± 961 for Psrg-13::gfp. p < 0.001, unpaired t-test. n = 20 neurons from two independent transgenic lines per genotype), which is consistent with our hypothesis as discussed in the Discussion section."

Moreover, Expression of slc-25A46 under the control of *srg-13* promoter could rescue the phenotype of slc-15A46 mutant (new Figure 1). Therefore, SLC-25A46 works as cell autonomously. SLC-25A46::mCherry was localized on mitochondria (new Figure 1F).

3) The present study offers little insight into the cell biological mechanisms of how SLC- 25A46 acts to promote mitochondrial fusion. The authors should make a prediction based on their data and test it experimentally. For example, what is the localization of FZO-1 in slc- 25A46 mutant animals compared to wildtype? Does FZO-1 colocalize with SLC-25A46?

We newly labelled endogenous FZO-1 by 3xgfp11 and analyzed the localization of FZO-1 in *slc-25A46* mutants. Unfortunately, we found that the amount of FZO-1 on mitochondria was not significantly affected (Below Figure). Because FZO-1 is a transmembrane protein, the localization may not be affected by *slc-25A46* mutations. Furthermore, as both FZO-1 and SLC25-A46 distributed on entire mitochondria, they colocalize on mitochondria.

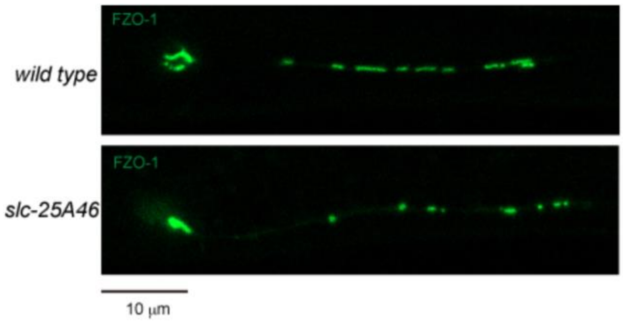

Therefore, we keep our discussion that SLC-25A46 may affect the GTPase activity of FZO-1. Biochemical assays are needed to conclude it. These are described in the Discussion section.

Minor Revisions:

4) The figures are incomplete and lack sufficient detail. In particular, the following areas must be addressed for completeness and clarity:

** Figure 2 has no quantifications and is unacceptable for publication in its current form. These phenotypes should be quantified. Furthermore, the dendrite of the slc-25A46; drp-1 double mutant needs to be shown in addition to the cell body. Please quantify and report the degree of rescue in mitochondrial number and size.*

We newly added quantification and showed dendritic phenotype of the double mutants (Figure 2).

** For the TEM in Figure S1, please adjust the brightness and contrast settings so that the images are not so dark. These images are not informative without quantifications. Please provide a quantification of these data (this could be size, shape, circularity, circumference, or number of cristae).*

For quantification by EM images, one need to perform 3D reconstitution. To quantify the size, we used fluorescent microscopy. The purpose of EM observation is to study whether the fine structure of mitochondria were affected or not. While we observed more than 30 mitochondria by EM, we have not noticed any defects in the fine structure of mitochondria as described in the manuscript.

** Figure 4 E and F lack labels on the Y axis.*

We corrected them. Now they are Figure 4B and C.

** Figure 4 E and F are very confusing to the reader. There is no way to know from the figure, or from the figure legend, that "Ex[slc-25a46]" refers to a fosmid with the slc-25a46 genomic DNA.*

© 2025. Published by The Company of Biologists under the terms of the Creative Commons Attribution License (https://creativecommons.org/licenses/by/4.0/).

(Figure S2 uses the same "Ex[slc-25a46]" nomenclature to refer to cDNA driven by the flp-15 promoter.) The only way to distinguish the two is by referencing the text. At first glance, Figures 4 and S2 appear to completely contradict each other. Please add labels to 4 E and F, as well as the figure legend, to clarify the use of a fosmid in this panel. Labels should be added to Figure S2 and its legend as well.

We tested three promoters (srg-13, flp-15 and osm-6), and noticed that slc-25A46 induces mitochondrial fragmentation when expressed under the control of either flp-15 or osm-6 promoter. Therefore, In the rescue experiments, we unified the promoter to *srg-13* (Figure 4).

For the fragmentation induced by the overexpression, we showed the result of *osm-6* promoter in new Figure 5.

** In Figure 4 E and F, please show the statistical significance between the array(-) and array(+) strains within the slc-25a46 and fzo-1 backgrounds.*

We newly analyzed our data (Figure 4).

5) The lack of phenotype enhancement in the slc-25a46; fzo-1 double mutant is one of the strongest pieces of evidence substantiating the claim that SLC-25A46 acts upstream of FZO-1. It is my opinion that this result should be reported in the main figure rather than in the supplement.

We moved the data to new Figure 4 and unified it with rescue data (new Figure 4).

6) The Methods section lacks sufficient detail. In particular, the following areas need to be addressed:

** How was mitochondrion size / area measured and quantified?*

We newly prepared " *Measurement of mitochondrial size and number*" section in the methods.

** What criteria were used to define and score fragmentation categories? This is especially important for the reader to evaluate the value of the data presented in Figure 5.*

We added following explanation in the methods section;

"For categorization of mitochondrial phenotypes in Figure 3C and 6D, mitochondrial morphology was visually classified into four categories shown in Figure 3B, based on fluorescent images. "Weakly fragmented" mitochondria were defined as consisting of predominantly short tubules with occasional discontinuities, whereas "highly fragmented" mitochondria appeared as numerous small, discrete puncta totally lacking tubular continuity. When each mitochondrial dots were not clear, the phenotype was classified as "aggregated"."

** How was "branch occupancy" measured in Figure 6, and what does this metric mean?*

We added following explanation:

"To quantify the increase in ectopic dendritic branches, we measured the relative area occupied by neuronal processes within a defined region surrounding the PVD cell body (Fig 7D). Specifically, we defined a 100 µm × Y µm rectangular area centered on the soma, where the Y-dimension was bounded by the adjacent tertiary branches (Fig 7A, grey area). This parameter was used instead of direct branch counting, as distinguishing individual ectopic branches from normal higher-order dendrites was ambiguous due to their irregular orientation and morphology, especially in later stages in mutants."

7) The text needs to be revised for completeness. The following areas should be addressed:

** Figure 1 C and D should be explicitly discussed in the text.*

These are Figure 1D and E in the revised manuscript. We described these data in the main text.

8) The title contains the phrase, "and is Essential for Maintaining Neuronal Morphology." While this phrase may be technically accurate from a genetics standpoint, this property is certainly not unique to SLC-25A46, and could be said of any number of proteins involved in mitochondrial transport, positioning and morphology. Furthermore, the role of SLC-25A46 is almost certainly quite a few steps upstream of neurodegeneration and neuronal morphology. It is my opinion that this phrase unnecessarily exaggerates the importance of the findings and should be removed from the title. In addition, since neuronal integrity was examined in a neuron in which the authors did not examine mitochondrial phenotypes, this conclusion is a bit misleading.

We newly observed mitochondrial morphology in PVD neuron and found that mitochondrial

© 2025. Published by The Company of Biologists under the terms of the Creative Commons Attribution License (https://creativecommons.org/licenses/by/4.0/).

morphology was affected in these neurons. We agree with the reviewer that the phenotype is not specific to *slc-25A46* mutants, but we think our results indicate the role of SLC-25A46 in the maintenance of neuronal morphology, which is consistent with the fact that mutations in human SLC25A46 is associated with neuronal diseases.

9) The authors report defects in the morphology of the PVD neuron in slc-25A46 mutants. I would suggest removing this figure unless mitochondria are also examined in this neuron.
We newly observed mitochondrial morphology in PVD neuron (new Figure S2).

10) It is unclear why nonparametric statistical tests were used exclusively throughout the paper. Were the data non-normally distributed? The data points visually do not appear to be so. The rationale for this choice should be explained in the Methods; otherwise, standard one-way ANOVAs with the mean and SD error bars should be plotted rather than the median. We have assessed the distribution of our datasets using visual inspection and considered the data were not normally distributed. Therefore, we chose to use nonparametric statistical tests to avoid type I errors in Figure 1 to 6. In Figure 7, as the data indicate normal distribution, parametric analysis were used.
This has now been clearly described in the Methods section.

Thank you very much for your advice.

Reviewer 2: Through unbiased forward genetic screening in C. elegans , Obinata et al discovered that SLC-25A46 is essential for maintaining mitochondrial and neuronal morphology. Their findings revealed that SLC-25A46 functions upstream of fzo-1 in regulating mitochondrial morphology. They also explored the impact of disease-related mutations in SLC-25A46 on mitochondrial and neuronal morphologies. Notably, their findings suggested that the expression level of SLC-25A46 influences mitochondrial morphology, which helps to explain inconsistencies in previous studies regarding its role in mitochondrial fission and fusion. Overall, the manuscript is well-written, and the data derived from a well- designed experiment are sound. However, some conclusions are not fully supported by the data. The following points should be addressed before publication in JCS.
Thank you very much for reading our manuscript.
Major concerns;
1. In Figure 1A, B, the mitochondrial signal appears saturated, and the images are too small to clearly depict mitochondrial morphology. Please provide unsaturated, magnified images of mitochondria in both cell bodies and dendrites.
We prepared new images showing mitochondrial morphology in the cell body and dendrites (Figure 1). Please note that it was difficult to show both cell body signal and dendritic signals together, and therefore, we showed each region separately.

2. The authors observe that the loss of SLC25A46 (jpn15) leads to a decrease in both the number of mitochondria per dendrite and the size of individual mitochondria (Fig. 1C and 1D). Inhibition of mitochondrial fusion would increase the number of mitochondria, similar to the eat-3 mutant phenotype shown in Fig. 3F and 3G. How do the authors explain the reduction in the number of mitochondria in dendrites of the SLC25A46 mutant?
We think intracellular transport may be affected by slc-25A46 mutations. It is now included in the discussion.
"*Furthermore, mitochondrial number is more severely reduced in slc-25A46 and fzo-1 mutants than in eat-3 mutants. The phenotype suggests an additional role for the outer membrane fission machinery in mitochondrial distribution. One possibility is that the dendritic transport of mitochondria is functionally linked to the outer membrane dynamics. Consistent with this, TRAK1, a motor adaptor that connects mitochondria to microtubule motors, physically associates with MFN2 (FZO-1 ortholog). Further investigation will be required to clarify how mitochondrial dynamics and trafficking are coordinated.*"
3. Single images from each mutant line are not sufficient to demonstrate the mitochondrial fragmentation phenotype in the cell body in Fig. 2A. As this phenotype is used as an indicator for identifying suppressor mutants of the jpn15 allele, it should be quantified and statistically analyzed.
We newly quantified the phenotype (new Figure 2).

© 2025. Published by The Company of Biologists under the terms of the Creative Commons Attribution License (https://creativecommons.org/licenses/by/4.0/).

4. The definition of "bead-like structures" is missing in Fig 6B. Clear definition of the bead- like structures used for quantification in Fig. 6C and arrows to indicate them in Fig. 6B, probably with higher magnification images, are required.
We clearly defined it.
"Bead-like structures, a reported sign of neuronal degeneration (Zhao et al., 2021)(Nakano et al., 2022)(Oren-Suissa et al., 2017), are defined in this study as regions where the dendrite diameter is more than three times that of the adjacent shaft. The number of such structures was manually counted along the dendrite in each neuron, revealing a significant increase in slc-25A46 mutants at day 1 and day 3 (Fig 7C)."

5. The authors showed that jpn15 and disease-related mutants exhibit abnormal neuronal morphology in PVD neurons. Is this phenotype accompanied by abnormal mitochondrial morphology? Mitochondria morphology should be investigated in PVD neurons.
We newly visualized mitochondria in PVD neurons (supplementary Figure S2). Similar to PHA neuron, the size was affected but the number was not affected.

Minor points;
1. On page 9 lines 1-2, "The mammalian SLC25A46 has been reported to physically interact with the outer and inner membrane fusion factors MFN1 and MFN2 (FZO-1 orthologs) (Steffen et al., 2017)." Does it mean MFN1 and MFN2 (or one of those) are inner membrane fusion factors as well? If not, an inner membrane fusion factor should be named here.
We corrected this mistake.

2. On page 9 line 17: Fig 4G instead of Fig 4D
We corrected it.

3. On page 10 lines 9-12: It appears that Fig 5F and Fig 5G have been swapped.
We corrected it.

4. Fig 6B: What do the asterisks in the images indicate? Please mention it in the figure legend.
Asterisks indicates possible ectopic branches.

5. The discussion regarding how the expression level of SLC25A46 can influence the phenotype of mitochondrial morphology is very informative.
Thank you very much.
However, the statement on page 11, line 19, "an observation in mutant mice considered to be hypomorph," which should refer to [Li et al., 2017, Hum. Mol. Genet. 26, 3776-3791, PMID: 28934388], appears to describe the original paper's claim incorrectly. What is the rationale for using the term "hypomorph"? In their paper, Li et al. created a transgenic knockout mouse by inducing a 46-bp deletion in exon 8 (C-terminal) and confirmed that endogenous Slc25a46 signals were undetectable in western blot analysis using antibodies targeting the N-terminal of Slc25a46 in the Slc25a46-/- cortex and cerebellum.
We corrected the discussion.
"The functional role of SLC25A46 remains controversial, as previous studies have reported inconsistent mitochondrial phenotypes across different models. Mitochondrial hyperfusion has been observed in knockdown models of zebrafish and Drosophila (Suda et al., 2018; Wan et al., 2016), while both hyperfusion and fragmentation have been reported in mammalian systems depending on the mode of genetic manipulation (Abrams et al., 2015; Janer et al., 2016; Li et al., 2017; Steffen et al., 2017). In our C. elegans model, the slc-25A46(jpn15) allele, which has an early stop codon and is likely a null mutation, exhibited mitochondrial fragmentation. These findings suggest that mitochondrial morphology in response to SLC25A46 loss may depend not only on the degree of gene disruption, but also on context-dependent factors."

Second decision letter

© 2025. Published by The Company of Biologists under the terms of the Creative Commons Attribution License (https://creativecommons.org/licenses/by/4.0/).

MS ID#: jcs.263571R1

MS Title: SLC-25A46 Regulates Mitochondrial Fusion through FZO-1/Mitofusin and is Essential for Maintaining Neuronal Morphology

Authors: Hiroyuki Obinata; Taisei Watanabe; Hironori Takahashi; Satoshi Shimo; Toshiyuki Oda; Asako Sugimoto; Shinsuke Niwa

Article Type: Research Article

Dear Dr Niwa,

I am happy to tell you that your manuscript has been accepted for publication in Journal of Cell Science, pending standard publication integrity checks.

Reviewer 1

Advance summary and potential significance to field

The authors addressed most of my concerns. I think that the paper is ready for publication.

SUGGESTIONS TO AUTHORS

© 2025. Published by The Company of Biologists under the terms of the Creative Commons Attribution License (https://creativecommons.org/licenses/by/4.0/).
